# Monocytes release cystatin F dimer to associate with Aβ and aggravate amyloid pathology and cognitive deficits in Alzheimer’s disease

**DOI:** 10.1186/s12974-024-03119-2

**Published:** 2024-05-10

**Authors:** Qiang Li, Bing Li, Li Liu, Kang-Ji Wang, Ming-Yue Liu, Yu Deng, Ze Li, Wei-Dong Zhao, Li-Yong Wu, Yu-Hua Chen, Ke Zhang

**Affiliations:** 1 Department of Developmental Cell Biology, Key Laboratory of Cell Biology,, Ministry of Public Health, China Medical University, 77 Puhe Road, Shenyang, 110122 China; 2 Department of Laboratory Medicine, Shengjing Hospital of China Medical University, Shenyang, 110004 China; 3 Department of Neurology, Xuanwu Hospital, Capital Medical University, Beijing, 100053 China; 4 Department of Neurology, Shenyang Fifth People Hospital, Shenyang, 110023 China; 5 Department of Environmental Health, School of Public Health, China Medical University, Shenyang, 110122 China

**Keywords:** Alzheimer’s disease, Amyloid beta, Cystatin F, Monocyte, Peripheral clearance

## Abstract

**Background:**

Understanding the molecular mechanisms of Alzheimer’s disease (AD) has important clinical implications for guiding therapy. Impaired amyloid beta (Aβ) clearance is critical in the pathogenesis of sporadic AD, and blood monocytes play an important role in Aβ clearance in the periphery. However, the mechanism underlying the defective phagocytosis of Aβ by monocytes in AD remains unclear.

**Methods:**

Initially, we collected whole blood samples from sporadic AD patients and isolated the monocytes for RNA sequencing analysis. By establishing APP/PS1 transgenic model mice with monocyte-specific cystatin F overexpression, we assessed the influence of monocyte-derived cystatin F on AD development. We further used a nondenaturing gel to identify the structure of the secreted cystatin F in plasma. Flow cytometry, enzyme-linked immunosorbent assays and laser scanning confocal microscopy were used to analyse the internalization of Aβ by monocytes. Pull down assays, bimolecular fluorescence complementation assays and total internal reflection fluorescence microscopy were used to determine the interactions and potential interactional amino acids between the cystatin F protein and Aβ. Finally, the cystatin F protein was purified and injected via the tail vein into 5XFAD mice to assess AD pathology.

**Results:**

Our results demonstrated that the expression of the cystatin F protein was specifically increased in the monocytes of AD patients. Monocyte-derived cystatin F increased Aβ deposition and exacerbated cognitive deficits in APP/PS1 mice. Furthermore, secreted cystatin F in the plasma of AD patients has a dimeric structure that is closely related to clinical signs of AD. Moreover, we noted that the cystatin F dimer blocks the phagocytosis of Aβ by monocytes. Mechanistically, the cystatin F dimer physically interacts with Aβ to inhibit its recognition and internalization by monocytes through certain amino acid interactions between the cystatin F dimer and Aβ. We found that high levels of the cystatin F dimer protein in blood contributed to amyloid pathology and cognitive deficits as a risk factor in 5XFAD mice.

**Conclusions:**

Our findings highlight that the cystatin F dimer plays a crucial role in regulating Aβ metabolism via its peripheral clearance pathway, providing us with a potential biomarker for diagnosis and potential target for therapeutic intervention.

**Supplementary Information:**

The online version contains supplementary material available at 10.1186/s12974-024-03119-2.

## Background

Alzheimer’s disease (AD) is the most common form of dementia and an age-related neurodegenerative disease characterized by extracellular amyloid plaques, intracellular neurofibrillary tangles, and neuroinflammation triggered by activated microglia and reactive astrocytes [1, 2]. Accumulating evidence supports the idea that an imbalance between the production and elimination of amyloid beta (Aβ) is a very early sign of AD pathogenesis [3, 4]. Familial AD (early-onset AD), which is caused by the overproduction of Aβ due to mutations in the amyloid precursor protein (APP) and PSEN1 (PS1) genes, accounts for only 1% of all AD cases [5, 6]. However, in sporadic AD (late-onset AD), which represents 99% of all AD patients, impaired Aβ clearance is thought to be the leading cause of Aβ accumulation. Metabolic labelling studies have shown that Aβ clearance is impaired while Aβ production remains unchanged in sporadic late-onset AD [7]. Therefore, exploring dysfunctional molecular and cellular mechanisms involved in the clearance of Aβ will enhance the understanding of AD pathophysiology and promote the development of new clinical interventions.

Aβ is the product of the proteolytic cleavage of APP and is mainly expressed in neurons [8, 9]. Traditionally, Aβ has been thought to be cleared primarily by cells in the brain [10, 11]. For instance, microglia, the major type of innate immune cell in the brain, can eliminate various forms of Aβ through cell-mediated phagocytosis and extracellular enzymatic degradation [12, 13]. However, convincing evidence has indicated that the peripheral system plays a role in the clearance of Aβ [14–17]. Approximately 40–60% of brain-derived Aβ is estimated to be transported to the peripheral system for clearance [17, 18]. Blood monocytes are the counterparts of microglia in the periphery [19, 20]. Monocytes/macrophages are vital members of the peripheral innate immune system and act as the first line of host defence through multiple effector functions [21]. Blood monocytes clear Aβ that is transported from the brain and slow the progression of AD [22]. Some studies have shown that monocytes are more effective than resident microglia at providing neuroprotection, regulating neuroinflammation, and clearing Aβ in AD [18, 19, 23–25]. However, monocytes derived from patients with AD display an unexplainable degree of phagocytic dysfunction; for example, macrophages from the majority of AD patients fail to transport Aβ into endosomes and lysosomes [25, 26], monocytes inefficiently clear Aβ from regions of the AD brain [27], and the expression of phagocytosis-related receptors was decreased in monocytes from AD patients [28]. Therefore, the determinants of Aβ elimination by monocytes may be potential interventional targets for the peripheral clearance of Aβ.

Cystatin F, encoded by the *Cst 7* gene, is a secreted protein that is specifically expressed in immune cells such as monocytes/macrophages, lymphocytes, and neutrophils in the peripheral circulation and exclusively in microglia in the central nervous system. Cystatin F is most likely involved in the immune response [29–34]. When cystatin F is synthesized or taken up intracellularly, it enters the endosome-lysosome compartment, where it acts as an endogenous inhibitor of cysteine proteases such as cathepsin L and C [35]. Recently, cystatin F has been regarded as a disease-associated microglia (DAM) signature in AD that regulates microglial phagocytosis via an unclear mechanism [36, 37]. However, the role of cystatin F, especially its secreted form in peripheral monocytes, remains largely unknown.

Here, we identified increased mRNA expression of cystatin F in monocytes isolated from patients with AD. Monocyte-derived cystatin F exacerbate Aβ deposition in the brain and cognitive impairment in APP/PS1 mice. Mechanistically, we found that cystatin F was released by monocytes as a dimer into the plasma and physically interacted with Aβ to inhibit its internalization by monocytes. High-level cystatin F dimers in plasma rapidly aggravate cognitive impairment in 5XFAD transgenic mice, suggesting that circulatory cystatin F inhibits peripheral Aβ clearance and leads to deteriorated Aβ deposition in the brain, providing us with a potential therapeutic target for the elimination of Aβ in the periphery.

## Methods

### Patients and clinical assessment

Age-matched controls and AD patients were recruited from Xuanwu Hospital of Capital Medical University and First Hospital of China Medical University (sTable 1). The National Institute of Neurological and Communication Disorders and the Stroke and Alzheimer’s Disease and Related Disorders Association (NINCDS-ADRDA) were used as the basis for the patient selection criteria, and patients with other types of dementia were excluded from this study [38]. All of the healthy control individuals had no substantial comorbidities that could impair brain function and no family history of dementia, and the patients with AD did not have immunological illnesses or vascular risk factors [39]. The Mini-Mental State Examination (MMSE), the Montreal Cognitive Assessment (MoCA) [40], and the Clinical Dementia Rating (CDR) [41, 42] were all used to measure cognitive function and the degree of impairment in all subjects. To evaluate object recognition memory, the Rey Auditory Verbal Learning Test (RAVLT) was used as described previously [43]. All participants (or their legal guardians) provided written informed consent.

### Isolation of plasma, monocytes, lymphocytes and neutrophils

Approximately 5 mL of peripheral whole blood was extracted from each individual by venipuncture. The plasma was extracted from the whole blood by centrifuging for 10 min at 800 ×*g*. The monocytes and lymphocytes were separated by using Dynabeads Isolation Reagent (Invitrogen, Carlsbad, CA, Cat No. 11145D;11149D) according to the manufacturer’s instructions. Neutrophils were isolated by using Percoll (Sigma Aldrich, MO, USA, Cat No. P1644) density gradient centrifugation.

### RNA sequencing and data analysis

The RNA of the primary monocytes from the AD patients and age-matched controls was extracted by using TRIzol reagent (Invitrogen, Carlsbad, CA, Cat No. 15596026CN) according to the instructions. RNA sequencing analysis was carried out by Biomarker Technologies Company (Beijing, China). For data analysis, the fold change (FC), an absolute fold change between the two groups for each transcript, was calculated when comparing the two groups with distinct profiles. A Student's t test was used to determine the statistical significance of the differences between groups. The significantly differentially expressed transcripts had a FC of at least 4 and p values less than 0.05. By dividing the total number of genes in the Gene Ontology (GO) category by the total number of differentially expressed genes, enrichment factors were computed. Gene Set Enrichment Analysis (GSEA) v2.0.14 software was used to perform GSEA analysis.

### Mice

APP/PS1 transgenic mice were obtained from China Medical University. 5 × FAD transgenic mice were generous gifts from Professor Chao Wang of Chongqing Medical University. Monocyte-specific human cystatin F-overexpressing transgenic mice were generated as described in previous reports [44, 45]. Briefly, the open reading frame (ORF) of human cystatin F was obtained and subcloned and inserted into pBluescript SK (-) under the control of the human CD68 promoter. The fragment was removed and injected into fertilized C57BL/6 mice to construct transgenic mice named Hmo-cys F^+^ mice. Hmo-cys F^+^ and APP/PS1 AD model mice were bred to produce APP/PS1/Hmo-cys F^+^ double transgenic mice. All transgenic lines were backcrossed to the C57BL/6J strain. Mice were housed under specific pathogen-free conditions on a 12 h light/12 h dark cycle with free access to water and food and no more than five mice per cage. The temperature and humidity were 18–29 °C and 45–55%, respectively. In this study, only male 9-month-old APP/PS1 mice and 3-month-old 5XFAD mice were included. All the animal experimental procedures were approved by the Animal Experimentation Ethics Committee of China Medical University (CMU20240091).

### Morris water maze (MWM) test

The spatial memory and learning abilities of the mice were assessed by the MWM test. One day before the experiment, the mice were allowed to become accustomed to the water maze (100 cm in diameter). The pool was filled with water that was rendered opaque, which was drained every day, and the temperature of the water was maintained at 19 °C to 22 °C. During the training period, the mice were permitted to swim freely for 60 s to locate the platform (9 cm in diameter), which was fixed at a depth of 1 cm below the surface of the water. Mice that did not find the platform were directed to it and allowed to rest for 30 s. The mice were trained four times per day for six days. On day seven, the platform was removed, and the swimming activity of each mouse, including the latency, number of platform crossings and velocity within 1 min, was recorded. All the processes were monitored by using a video camera mounted overhead and automatically recorded via ANY-maze behavioural tracking software (Stoelting, Wood Dale, IL, USA).

### Novel object recognition (NOR) test

The NOR test was conducted as described previously [46]. Initially, the mice were acclimated to the testing room for five days before testing. Then, the mice were allowed to explore freely in the empty arena (40 × 40 × 40 cm) for 10 min. For the training session, two identical cylinders (6 cm in diameter) were placed in the arena 5 cm away from the wall. The mice were individually placed in the arena and allowed to freely explore for 10 min. Twenty-four hours after the training session, the NOR test was carried out. One of the familiar cylinders was replaced with a cube (with a side length of 6 cm). The mice were allowed to explore for 10 min, and a camera tracking system (TopScan Suite, CleverSys Inc., Reston, VA, USA) was used to record the amount of time the mice spent exploring the objects. The recognition index = time spent exploring the new object/(time spent exploring the new object + time spent exploring the familiar object) × 100%.

### Tail intravenous injections

Three-month-old 5xFAD mice were given tail intravenous injections of murine cystatin F dimer protein at a dose of 200 μg/kg every three days for one month. The experiments were performed 48 h after the last injection.

### Tissue preparation and immunofluorescence

Briefly, mice were transcardially perfused with phosphate-buffered saline (PBS) followed by ice-cold 4% paraformaldehyde (PFA) in PBS (pH 7.4). After perfusion, the brain was removed and postfixed in 4% PFA in PBS at 4 °C overnight and then transferred to 30% sucrose for 2-3 days until it sank to the bottom of the container. Coronal sections of the brain (30 μm) were cut using a cryostat (Minux FS800A, RWD Inc., China), collected serially on coated glass slides, and stored at − 20 °C until use. Six sections obtained from each brain containing the cortex and CA1 area of the hippocampus were chosen for immunofluorescence staining. The brain sections were incubated with PBS containing 0.5% Triton X-100 for 10 min and 5% BSA for 1 h at room temperature. The brain sections were sequentially incubated overnight at 4 °C with primary antibodies recognizing Aβ (1:800; Cell Signaling Technology, MA, USA, Cat No. 8243S), His tag (1:500; ABclonal Technology, Wuhan, China, Cat No. AE003) and IBAI (1:1000; Abcam, Cambs, UK, Cat No. ab178846). Then, the sections were incubated with Alexa Fluor 488-conjugated donkey-anti-rabbit IgG (1:200; Invitrogen, CA, USA, Cat No. A-21206) for 3 h in the dark at room temperature. After counterstaining with 4′,6-diamidino-2-phenylindole (DAPI) (Sigma Aldrich, MO, USA, Cat No. 28718-90-3) for 5 min, the coverslips were mounted and the slides were analysed under a laser scanning confocal microscope (LSCM).

### Enzyme-linked immunosorbent assay (ELISA)

The levels of soluble and insoluble Aβ in the prefrontal cortex were measured by ELISA method. Briefly, fresh brain tissues from the mice were homogenized in RIPA buffer [50 mM Tris (pH 7.4), 150 mM NaCl, 1% NP-40, and 0.5% sodium deoxycholate] supplemented with protease inhibitor cocktail, followed by centrifugation at 12,000 × g for 30 min at 4 °C. The supernatant was collected as the RIPA fraction, and the pellet was solubilized in 2% SDS and 25 mM Tris-HCl (pH 7.4) followed by centrifugation at 12,000 × g for 30 min at 4 °C as the SDS fraction. The concentrations of Aβ40 and Aβ42 were measured using ELISA kits (Elabscience Biotechnology Company, Wuhan, China, Cat No. E-EL-M3009; E-EL-M3010) according to the manufacturer’s instructions. The Aβ40 and Aβ42 in human plasma were also determined by the ELISA kits (Elabscience Biotechnology Company, Wuhan, China, Cat No. E-EL-H0542; E-EL-H0543). In addition, the levels of cystatin F in the plasma of patients with AD or AD mice were quantitatively analysed by ELISA kits (CUSABIO Company, Wuhan, China, Cat No. CSB-E17504h; CSB-EL006095MO). The data were normalized to the total protein concentration.

### Real-time polymerase chain reaction (RT-qPCR)

M-MLV reverse transcriptase was used to reverse transcribe the total RNA from the cells. RT-qPCR was conducted using a SYBR Premix Ex Taq Kit (Vazyme,Nanjing,China, Cat No. Q321-02) and an ABI 7500 RT-PCR instrument. Glyceraldehyde-3-phosphate dehydrogenase (GAPDH) was selected as the reference gene. The conditions for PCR were 95 °C for 10 s, followed by 40 cycles of 95 °C for 30 s and 60 °C for 30 s. Standard curves were established to calculate target gene transcript quantities in each sample.

### Western blot analysis

The plasma protein concentrations were determined by using a nondenaturing gel. Briefly, the plasma was diluted with 1X PBS, and nondenaturing loading buffer (50% glycerol, 0.3 mol/L pH = 6.8 Tris-HCl, 10% SDS, 0.01% bromophenol blue) was added to the samples. To detect the monomeric form of cystatin F, loading buffer with 20% dithiothreitol (DTT) (Sangon biotech, shanghai, China, Cat No. A620058) was used, and the samples were boiled at 95 °C for 10 min. Coomassie Brilliant Blue (CBB) staining of the plasma proteins served as an internal control. Cells were lysed with radioimmunoprecipitation assay lysis buffer (Beyotime Biotech, Beijing, China, Cat No. P0013B) containing 1 mM PMSF (Beyotime Biotech, Beijing, China, Cat No.ST506). Then, the protein samples were separated on 12% SDS polyacrylamide gels. For western blot detection, the primary antibody against cystatin F (ABclonal Technology, Wuhan, China, Cat No. A8164) was diluted 1:1000, and Horseradish Peroxidase (HRP) goat anti-rabbit IgG (ABclonal Technology, Wuhan, China, Cat No. AS014) was diluted 1:10,000. Immunoreactive bands were visualized with the SuperSignal West Pico chemiluminescent substrate (Pierce, IL, USA) using an LAS-3000 mini (Fujifilm, Tokyo, Japan). For quantitative analysis, the mean density of each band was measured by ImageJ software.

### Recombinant protein expression and purification

The ORF of cystatin F was subcloned and inserted into the pcDNA3.1 (+) myc-His A vector, and the recombinant plasmids were transfected into 293T cells with polyethylenimine (PEI) (Polysciences, IL, USA, Cat No. 02371-500) at a ratio of 1 µg of DNA to 2.5 µg of PEI [47, 48]. The cell culture supernatant was collected after 48 and 72 h of transfection, and the secreted cystatin F protein was purified by using His-tag Purification Resin (Beyotime, Shanghai, China, Cat No. P2210).

### Aβ internalization assay

The Aβ internalization by monocytes were assessed by flow cytometry analysis (FCM), ELISA and LSCM methods. For FCM and ELSIA assay, 3 × 10^5^ cells per well were cultured in 24-well plates, and the cystatin F dimer protein was added at final concentrations of 0 ng/mL, 50 ng/mL, 250 ng/mL, and 1000 ng/mL for 30 min. Then a final concentration of 1 μg/mL Aβ42-Alexa Fluor 647 (Kaneka Eurogentec, Liege Province, Belgium, Cat No. AS-64161) or soluble Aβ42 was added for a further incubation for 30 min at 37 °C, after which the cells were subjected to FCM and ELISA analysis, respectively. For LSCM analysis, cells were pretreated with the cystatin F dimer at a final concentration of 250 ng/mL for 30 min and then cells were incubated with Aβ42-Alexa Fluor 555 (Kaneka Eurogentec, Liege Province, Belgium, Cat No. AS-60480-01) for an additional 30 min at 37 °C. To analysis the binding ability of cystatin F dimer to Aβ at the cell membrane by FCM and ELISA methods, cells were pretreated with the cystatin F dimer at a final concentration of 250 ng/mL for 30 min at 37 °C, and then cells were incubated with Aβ42-Alexa Fluor 647 or soluble Aβ42 for an additional 30 min at 0 °C, after which the cells were subjected to FCM and ELISA analysis, respectively. For LSCM analysis, cells were pretreated with the cystatin F dimer at a final concentration of 250 ng/mL for 30 min at 37 °C, and then cells were incubated with Aβ42-Alexa Fluor 555 for an additional 30 min at 0 °C. The coverslips were labelled with 15 μg/mL 3,3′-dioctadecyloxacarbocyanine perchlorate (DiOC18(3), Beyotime, Shanghai, China, Cat No. C1038) in 4% PFA solution for 15 min, and stained with DAPI at a final concentration of 1 μg/mL for 4 min.

For the uptake inhibition assay, cytochalasin D (GLPBIO, CA, USA, Cat No. D22914GC13440) or EIPA (MedChemExpress, NJ, USA, Cat No. HY-101840) at final concentrations of 5 μg/mL or 40 μM were added to the cells to pretreat for 30 min and 1 h, respectively. The cells were incubated with the cystatin F dimer at a final concentration of 250 ng/mL for 30 min. Aβ42-Alexa Fluor 647 at a final concentration of 1 μg/mL was added to the cells, which were incubated for 30 min and analysed via FCM method. The data were analysed with InCyte software (Millipore, Darmstadt, Germany) and visualized using FlowJo software (Tree Star, Inc., CA, USA).

### Total internal reflection fluorescence microscopy (TIRFM)

Monocytes were cultured on coverslips, and after treatment with 250 ng/mL cystatin F protein for 30 min, the cells were incubated with 1 μg/mL soluble Aβ42-Alexa Fluor 555 for 30 min at 0 °C and washed three times with PBS. The monocytes on the coverslips were fixed with 4% PFA solution for 15 min and were analysed under a TIRFM.

### Binding assay

The 96-well ELISA plates were coated with 4 ng/μL of dissolved Aβ1-40, Aβ1-42, Aβ40-1, or Aβ42-1 for 16 h at 4 °C. Bull serum albumin at a 5% concentration was used as a blocking agent and incubated for 2 h at 37 °C. Recombinant cystatin F dimer protein was added to the plates for 3 h at various concentrations (18.75 ng/mL, 37.5 ng/mL, 75 ng/mL, 150 ng/mL, and 300 ng/mL). Cystatin F antibody (R&D Systems, MN, USA, Cat No. MAB1889) was used to identify the binding of the two proteins. A secondary antibody coupled to HRP and 3,3′,5,5′-Tetramethylbenzidine reagent was used to induce a colour reaction, and the absorbance was read at 450 nm.

### Molecular docking

Protein Aβ42 (PDB ID: 6SZF) and cystatin F dimer (PDB ID: 2CH9) crystal structures were retrieved from the RCSB Protein Data Bank (http://www.rcsb.org/). The protein cystatin F was set as the receptor before docking, while Aβ42 was set as the ligand. Cluspro1, HDOCK2, and MOE3 were utilized to determine the binding mechanism of Aβ42 and the cystatin F dimer using three molecular docking tools.

### Pull-down assay

Purified GST-Aβ protein (Abcam, Cambridge, UK) and His-tag cystatin F were co-incubated overnight at 4 °C. The purification resin (Beyotime, Shanghai, China, Cat No. P2251; P2210) for the GST-tag or His-tag was added to the protein mixture and incubated for 2 h at 4 °C. The resins were centrifuged for 5 min at 1000 ×*g* and the precipitate was mixed with loading buffer, then the samples were subjected to Western blot analysis. Rabbit anti-His-tag and mouse anti-GST-tag (ABclonal Technology, Wuhan, China, Cat No. AE086; AE001) were diluted 1:1000. Goat anti-rabbit IgG or goat anti-mouse IgG conjugated with HRP was diluted 1:10,000 (ABclonal Technology, Wuhan, China, Cat No. AS070; AS066).

### Bimolecular fluorescence complementation assay (BiFC)

The BiFC was carried out as previously reported with modifications [49]. Briefly, full-length ORF of cystatin F conjugated with the N-terminal fragment Venus and of Aβ conjugated with the C-terminal fragment Venus were constructed. The recombinant plasmids were transfected into 293T cells, and the protein supernatants were purified. The two proteins were incubated at 200 ng/mL for 2 h at 37 °C, and the fluorescence intensity was detected at 529 nm.

### Statistical analysis

Data were presented as mean ± standard deviation (SD). Student’s t test was used to compare two groups while one-way ANOVA was used to compare more than two groups. A receiver operating characteristic (ROC) curve was generated to evaluate the diagnostic value of the cystatin F in AD monocytes. Correlations were assessed by using Pearson correlation coefficient. All data were analysed by GraphPad Prism 8.0 statistical software. Statistical significance was indicated by a probability (p) value of less than or equal to 0.05.

## Results

### Elevated cystatin F expression in monocytes isolated from patients with AD

**Fig. 1:**
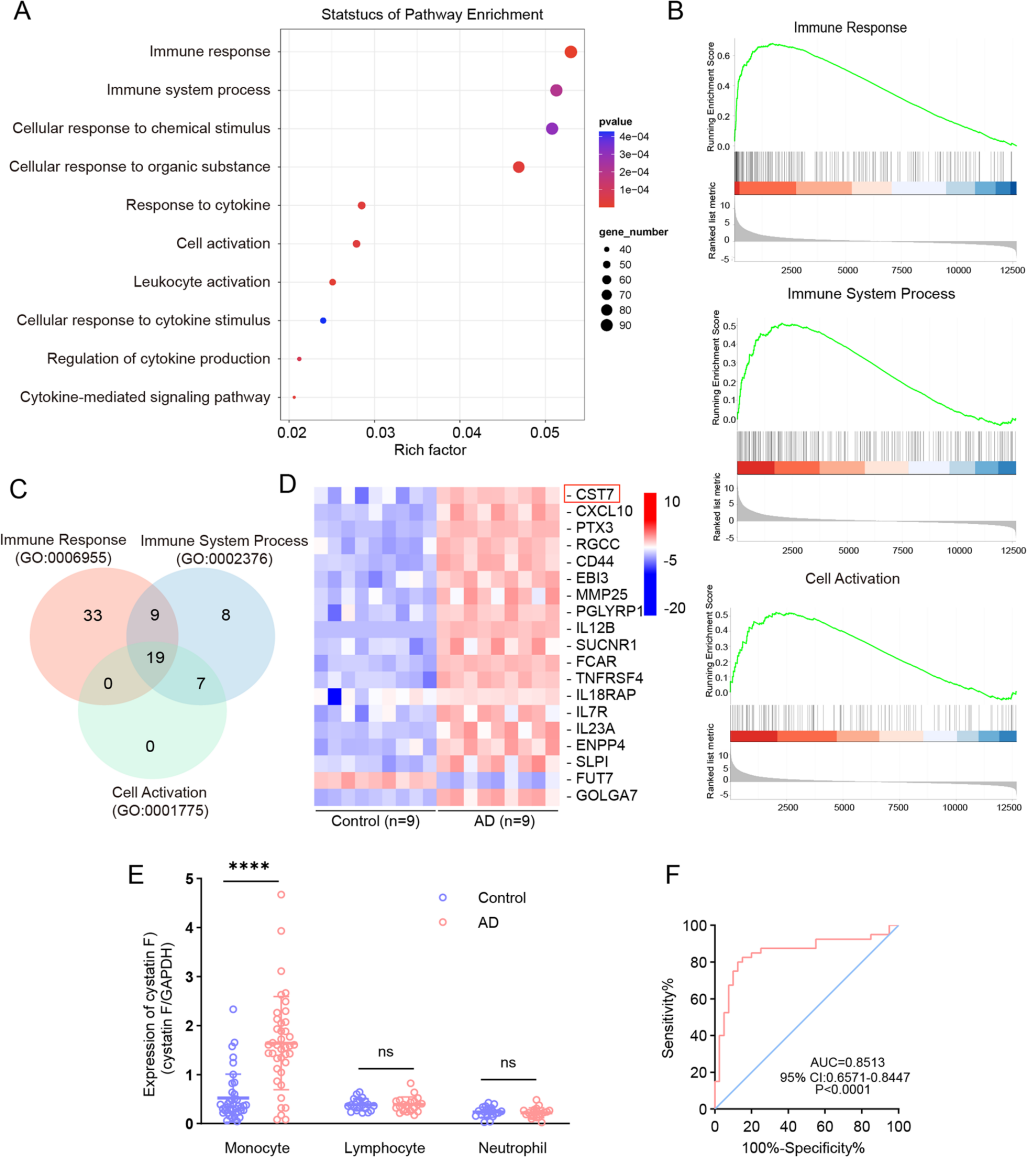
The mRNA expression of cystatin F was significantly increased in the monocytes of patients with AD. Monocytes derived from AD patients (n = 9) and age-matched controls (n = 9) were collected for RNA-sequencing analysis. **A** GO enrichment analysis of biological processes showing the top 10 signalling pathways. **B** GSEA enrichment analysis of genes co-expressed in the three GO pathways. **C** Venn diagram of the genes co-expressed in the three GO pathways. **D** Heatmap showing the 19 differentially expressed genes. **E** The mRNA expression of cystatin F in monocytes from AD patients (n = 40) and controls (n = 40), and in lymphocytes (n = 20) and neutrophils (n = 20) from AD patients and controls (n = 20) was measured by RT-qPCR. **F** ROC curve analysis of cystatin F mRNA expression in monocytes from AD patients. The data are presented as the means ± SD and were analysed using Student’s t test. ^****^p < 0.0001

First, we collected samples from sporadic AD patients and isolated monocytes for RNA-seq analysis (sTable 1 and sFig. 1A). We performed a  GO functional enrichment analysis of the differentially expressed genes with four fold changes in expression (Fig. 1A and sFig. 1B and C). Since monocytes are the main peripheral innate immune cells involved in the immune response and clearance of pathogenic agents, we selected the GO terms “immune response” (GO:0006955), “immune system process” (GO:0002376), and “cell activation” (GO:0001775) from the top 10 GO pathways to perform a GSEA analysis of these differentially expressed genes (Fig. 1B). There were 19 core enriched terms in the three pathways (Fig. 1C and D). Among them, only four enrichments were specific to immune cells (sTable 2). Ultimately, we chose the cystatin F protein as a target because it is expressed in myeloid leukaemia cells involved in the innate immune response (Fig. 1D and sTable 2). Cystatin F also has been reported to be expressed in microglia, which are central innate immune cells [50, 51]. Next, we examined the mRNA level of cystatin F in monocytes isolated from AD patients by RT-qPCR with an expanded sample size (n = 40; sTable 1). The results showed that the mRNA expression of cystatin F was obviously greater in the AD group than in the elderly control group (Fig. 1E). Neutrophils and lymphocytes are the main types of immune cells that participate in innate and adaptive immune responses, respectively. We also examined the mRNA expression of cystatin F in the two types of immune cells. The results indicated that the transcript level was not significantly different between the AD group and age-matched controls (Fig. 1E). Furthermore, the specificity and sensitivity of the mRNA expression of cystatin F in AD monocytes were analysed, and ROC analysis indicated that the area under the curve (AUC) reached 0.8513, which was statistically significant (Fig. 1F). These results demonstrated that aberrant expression of cystatin F principally occurs in AD monocytes.

### Overexpression of human cystatin F in monocytes aggravated Aβ deposition and cognitive decline in APP/PS1 mice

**Fig. 2:**
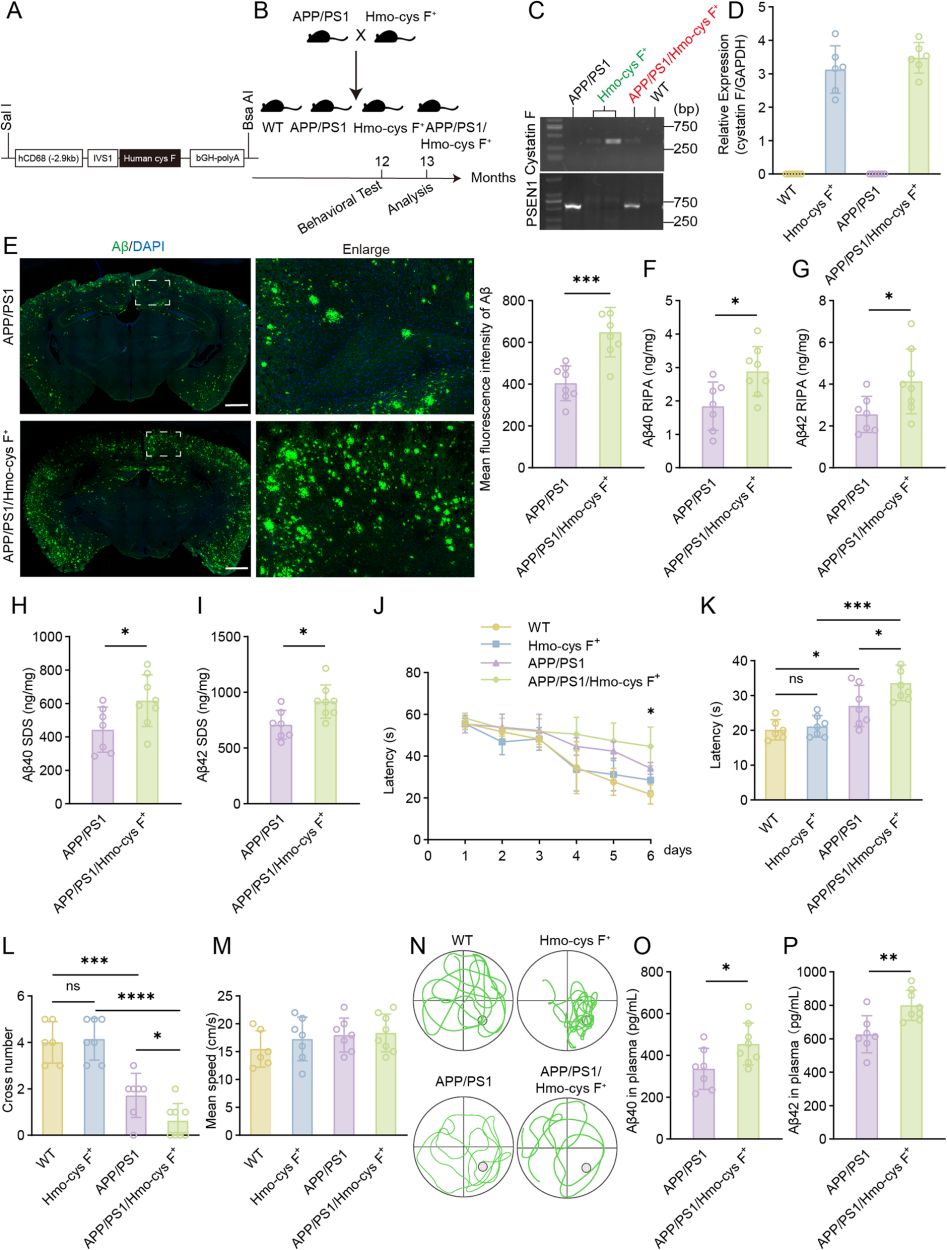
Monocyte-specific overexpression of human cystatin F exacerbated cognitive impairment in APP/PS1 transgenic mice.** A** Construction of monocyte-specific overexpression of human cystatin F under the control of the human CD68 promoter. **B** Schematic for generating APP/PS1/Hmo-cys F^+^ mice, behavioural tests, and pathological analysis. **C** Identification of transgenic mice by PCR analysis of the genomic DNA. **D** Transcript levels of human cystatin F in the monocytes of 6-month-old WT (n = 6), Hmo-cys F^+^ (n = 6), APP/PS1 (n = 6), and APP/PS1/Hmo-cys F^+^ mice (n = 6). **E** Immunofluorescence and statistical analysis of Aβ plaques in brain sections from transgenic mice. Scale bar: 1000 μm **F**, **G** ELISA analysis for soluble **F** Aβ40 and **G** Aβ42 levels in brains extracted with RIPA buffer. **H**, **I** ELISA analysis for insoluble **H** Aβ40 and **I** Aβ42 levels in brains extracted with SDS buffer. **J**, **K** MWM analysis showing the latency (s), **L** number of target crosses, and **M** mean speed (cm/s) in the invisible platform test. **N** Representative images of the track plots in the MWM test.** O**, **P** ELISA analysis of **O** Aβ40 and **P** Aβ42 levels in the plasma of mice. All the mice in the behavioural test and Aβ analysis were 12 months old and included WT (n = 6), Hmo-cys F^+^ (n = 7), APP/PS1 (n = 7), and APP/PS1/Hmo-cys F^+^ mice (n = 8). The data are presented as the means ± SD and were analysed using Student’s t test and one-way ANOVA. ^*^p < 0.05, ^**^p < 0.01, ^***^p < 0.001, ^****^p < 0.0001

To explore the role of cystatin F in monocytes in the context of AD, we generated transgenic mice on the C57BL/6 background with monocyte-specific expression of human cystatin F under the human CD68 promoter (Hmo-cys F^+^) (Fig. 2A) [44, 45]. Hmo-cys F^+^ mice were hybridized with APP/PS1 mice to obtain APP/PS1/Hmo-cys F^+^ mice via double transgenics to mimic the high cystatin F levels observed in the monocytes of AD patients (Fig. 2B-D). First, we detected Aβ deposition and measured the Aβ concentration in the brain. The Aβ plaque coverage within the brains of APP/PS1/Hmo-cys F^+^ mice were markedly greater than that within the brains of APP/PS1 mice according to immunofluorescence staining (Fig. 2E). Additionally, there was a significant increase in soluble Aβ40 and Aβ42 levels (Fig. 2F, G) and insoluble Aβ40 and Aβ42 levels (Fig. 2H, I) in the brains of APP/PS1/Hmo-cys F^+^ mice. Furthermore, we assessed the spatial learning and memory of the mice through the MWM test. Compared with APP/PS1 mice, APP/PS1/Hmo-cys F^+^ mice showed significant spatial learning and memory deficits, as reflected by longer escape latency time during the training trials and a lower number of platform crossings (Fig. 2J-L, N). However, there was no significant alterations in swimming speed (Fig. 2M). In addition, the levels of soluble Aβ40 and Aβ42 were significantly increased in the plasma of APP/PS1/Hmo-cys F^+^ transgenic mice compared with APP/PS1 mice (Fig. 2O, P). These results suggested that human cystatin F specifically aggravated Aβ deposition in the brain and cognitive impairment in AD model mice.

### Increased cystatin F dimer levels in the plasma of AD patients

**Fig. 3:**
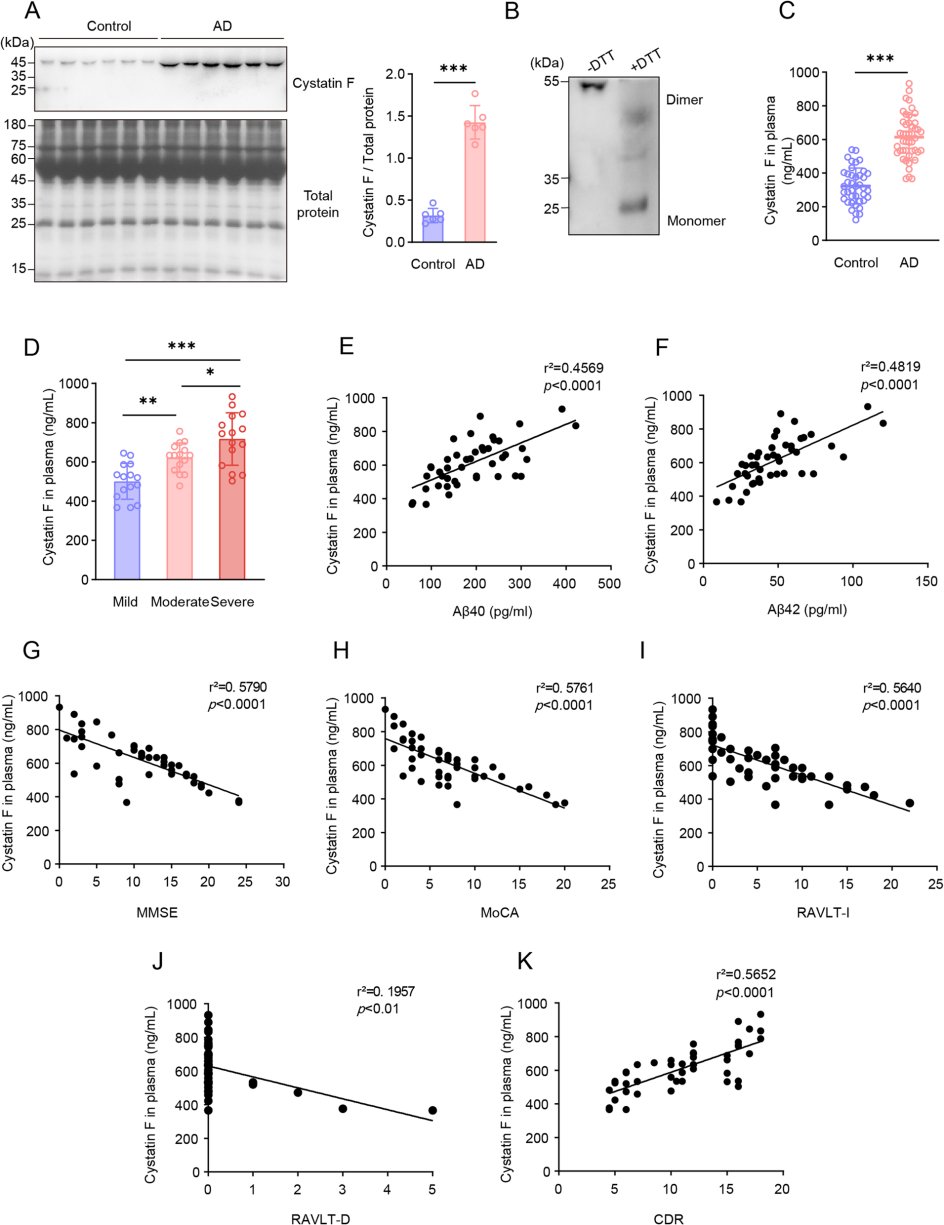
Identification of the expression and structure of the cystatin F protein in the plasma of AD patients and analysis of its correlation with AD clinical manifestations. **A** Western blot analysis of the expression and structure of the cystatin F protein in plasma from patients with AD (n = 6) and controls (n = 6) by using a nondenaturing gel. The total protein in the plasma was stained with CBB as an internal control. **B** The structure of cystatin F in human plasma was determined by western blot assay, and DTT was added to the sample to reduce the dimers to the monomers. **C** ELISA analysis of secreted cystatin F levels in plasma from patients with AD (n = 45) and controls (n = 40). **D** ELISA analysis of secreted cystatin F in plasma according to the AD stage from mild (n = 15) to moderate (n = 15) to severe dementia (n = 15).** E**, **F** Correlations of the cystatin F level with the Aβ40 and Aβ42 levels in the plasma of patients with AD by Pearson correlation coefficient analysis. **G-K** Correlations of cystatin F levels with MMSE, MoCA, RAVLT-I, RAVLT-D and CDR scores by Pearson correlation coefficient analysis. The data are presented as the means ± SD and were analysed using Student’s t test and one-way ANOVA. ^*^p < 0.05, ^**^p < 0.01, ^***^p < 0.001

Mature cystatin F, which contains a signal peptide, is synthesized, and secreted into the extracellular space of cultured cells [50, 51]. Plasma is a major extracellular fluid makes up 55% of the peripheral blood, and the presence of cystatin F in plasma has not yet been investigated. Therefore, we used a nondenaturing gel to analyse the expression of cystatin F in the plasma of patients with AD. As shown in Fig. 3A, the level of cystatin F, which is approximately 50 kDa in molecular weight (MW), in the plasma from patients with AD was significantly greater than that in the plasma from controls. Cystatin F dimerizes through two disulfide bridges involving Cys26 on one subunit and Cys63 on the other [35]. We selected DTT, a reducing agent that can disrupt disulfide bonds to reduce dimers to monomers, to determine the structure of cystatin F in plasma. We found that the MW of cystatin F changed to approximately 25 kDa when DTT was added, suggesting that cystatin F in human plasma is a dimer in the physiological state (Fig. 3B). Furthermore, we examined the level of native cystatin F in cultured monocytes. Using a nondenaturing gel, the results showed that the intracellular cystatin F protein remains predominantly dimeric with few monomers and the extracellular proteins remained absolutely dimeric (sFig. 2). This finding suggested that the level of secreted cystatin F significantly increased in AD plasma as dimeric structure.

Next, we used ELISA to analyse the level of secreted cystatin F in the AD plasma with expanded cases, showing that cystatin F is significantly higher than that in age-matched controls (Fig. 3C), in accordance with the changes in its transcript levels. As the disease progressed, plasma cystatin F levels progressively increased depending on the AD stages (Fig. 3D). Furthermore, we examined the correlation between the cystatin F dimer concentration and the plasma Aβ40 or Aβ42 concentration. The results showed that the cystatin F dimer concentration was significantly positively correlated with the Aβ40 or Aβ42 concentration (Fig. 3E, F). These findings indicated that cystatin F dimer levels were correlated with Aβ levels. We also attempted to analyse the relationship between the cystatin F dimer concentration and the clinical manifestations of AD. The MMSE, MoCA, RAVLT-I, RAVLT-D, and CDR are important tools that provide complete and accurate information about mental health and cognitive status [40, 52]. Correlation analysis revealed that the plasma cystatin F concentration in patients with AD was significantly negatively correlated with the MMSE, MoCA, RAVLT-I, and RAVLT-D scores and positively correlated with the CDR (Fig. 3G-K). These results implied that high levels of cystatin F dimers in the plasma of patients with AD are associated with Aβ levels, the severity of AD, and the clinical features of AD.

### Extracellular cystatin F dimer inhibits Aβ phagocytosis by monocytic cells

**Fig. 4:**
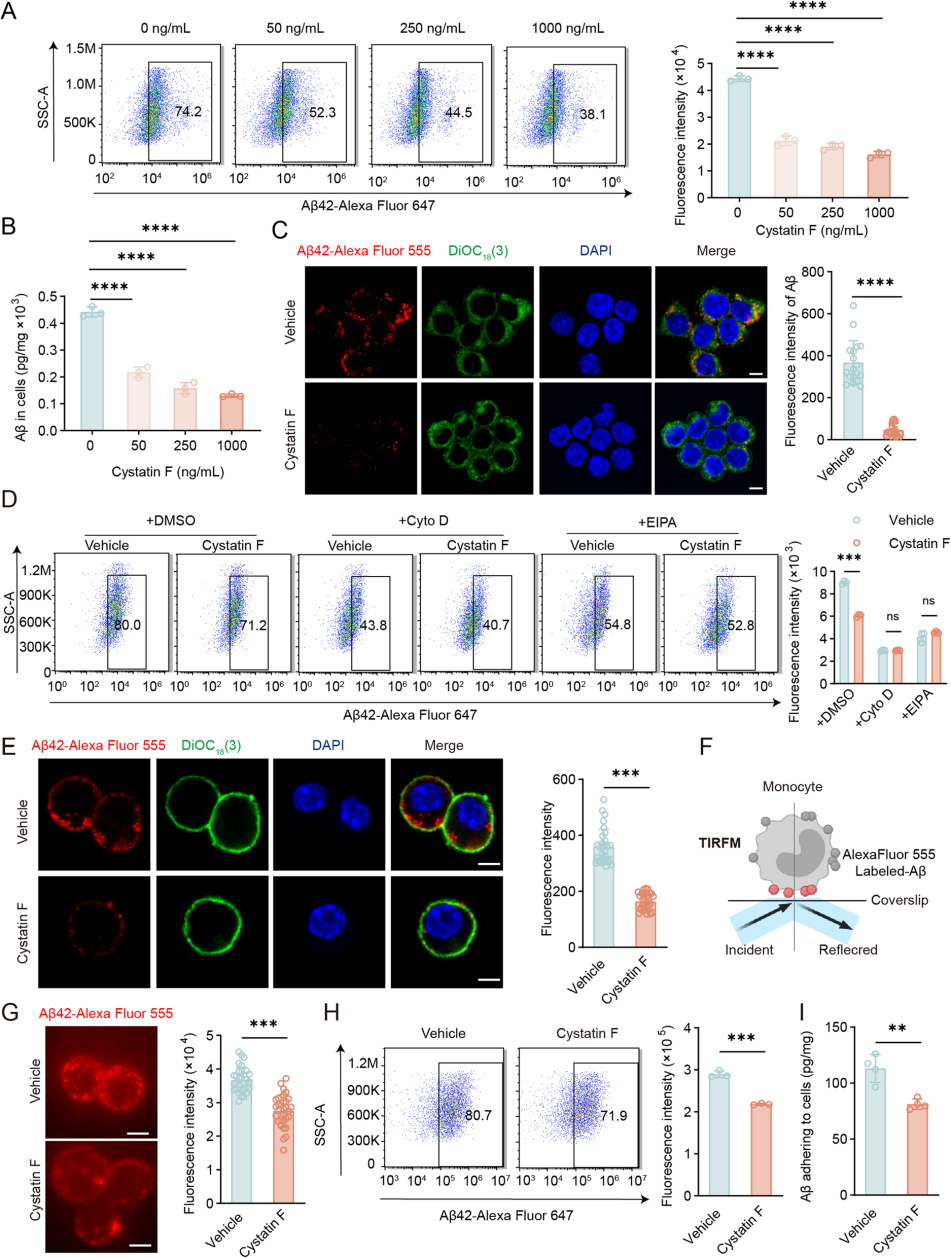
Cystatin F dimers inhibited the uptake of Aβ by monocytes. **A**-**C** THP-1 cells were pretreated with cystatin F dimer protein for 30 min, and 1 μg/mL soluble Aβ42-Alexa Fluor 647, or Aβ42, or Aβ42-Alexa Fluor 555 were added for an additional 30 min. Then, the cells were subjected to **A** FCM, **B** ELISA and **C** LSCM analysis. **D** THP-1 cells were pretreated with 5 μg/mL cytochalasin D for 30 min, 40 μM EIPA for 1 h, and 250 ng/mL cystatin F for 30 min. Then, cells were incubated with 1 μg/mL soluble Aβ42-Alexa Fluor 647 for 30 min and analysed by FCM method. **E** THP-1 cells were pretreated with 250 ng/mL cystatin F, and 1 μg/mL soluble Aβ42-Alexa Fluor 555 was added, then cells were incubated for 30 min at 0 °C. Then, the cells were observed by LSCM. **F**, **G** Schematic diagram depicting the use of TIRFM for imaging membrane-localized proteins. THP-1 cells were pretreated with 250 ng/mL cystatin F dimer for 30 min, and then 1 μg/mL soluble Aβ42-Alexa Fluor 555 was added and incubated for 30 min at 0 °C. Cells were observed by TIRFM. **H**, **I** THP-1 cells were pretreated with 250 ng/mL cystatin F, and 1 μg/mL soluble Aβ42-Alexa Fluor 647 or Aβ42 was added, then cells were incubated for 30 min at 0 °C. Then, the cells were subjected to** H** FCM and **I** ELISA. The data are presented as the means ± SD and were analysed using Student’s t test and one-way ANOVA. ^**^p < 0.01, ^***^p < 0.001, ^****^p < 0.0001. Scale bar: 5 μm

Given that monocytes/macrophages are crucial phagocytic cells in the innate immune system, we first investigated whether high levels of the cystatin F dimer impact the internalization of Aβ by monocytes. By the addition of purified cystatin F dimer protein to cultured monocytes to mimic the high concentration of cystatin F in the plasma of AD patients, we found that the uptake of Aβ42-AlexaFluor 647 by the monocytic cell line THP-1 gradually decreased with increasing concentrations of the cystatin F dimer via FCM analysis (Fig. 4A). The ELISA data showed that the intracellular Aβ42 levels decreased in a dose-dependent manner as the cystatin F dimer concentration increased (Fig. 4B). Additionally, we observed less Aβ42-Alexa Fluor 555 in THP-1 cells pretreated with 250 ng/mL cystatin F dimer protein by LSCM (Fig. 4C). Notably, when the substrates were changed to fluorescence-labelled transferrin or dextran, monocytes exhibited similar levels of phagocytosis in the presence and absence of cystatin F (sFig. 3A and B), indicating that cystatin F inhibits Aβ phagocytosis by monocytes with a certain specificity. The process of internalization by phagocytic cells is known to be actin dependent [53]. We further selected cytochalasin D and EIPA, which are specific inhibitors of phagocytosis and macropinocytosis, respectively, to investigate the effect of the cystatin F dimer on intracellular phagocytic signalling [54, 55]. As shown in Fig. 4D, the two inhibitors effectively hindered the internalization of Aβ by monocytes; moreover, the suppressive effect was apparently reversed specifically by the cystatin F dimer protein, whereas cystatin F failed to reverse the inhibition of Aβ internalization as determined by the amount of fluorescently labelled dextran (sFig. 3C).

The recruitment of exogenous substances by monocytes is a prerequisite for phagocytosis. LSCM showed a significant reduction in Aβ42-Alexa Fluor 555 adhesion to the monocyte membrane when the cells were incubated in advance with the cystatin F dimer at 250 ng/mL (Fig. 4E). In addition, we used total TIRFM to observe the enrichment of Aβ42-Alexa Fluor 555 on the cell membrane of monocytes. The images showed that less Aβ42-Alexa Fluor 555 adhered to the surface of monocytes which were pretreated with purified cystatin F dimer protein (Fig. 4F, G). Moreover, co-incubating the substrate with cells at a lower temperature effectively prevented its internalization, showing that the cystatin F dimer was able to markedly inhibit the adhesion of Aβ to monocytes at 0 °C, as determined by FCM and ELISA analysis (Fig. 4H, I). These results demonstrated that the cystatin F dimer blocks monocyte phagocytosis by hindering the binding of Aβ to the monocyte membrane.

### Cystatin F dimer physically interacts with Aβ to inhibit its internalization by monocytes

**Fig. 5:**
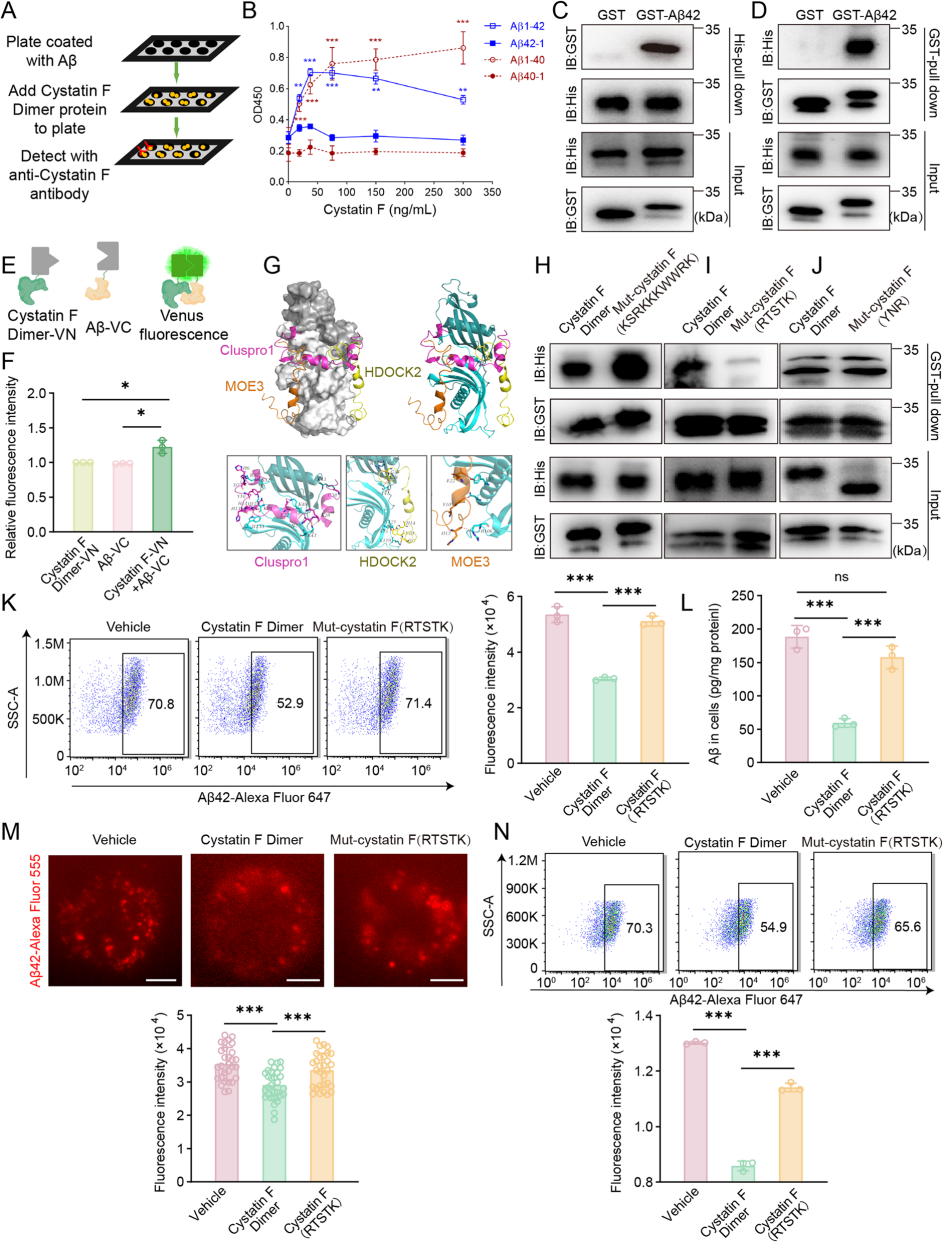
Cystatin F dimers physically interact with Aβ to inhibit the internalization of Aβ by monocytes.** A** Schematic of the binding assay. **B** Plates were coated with 4 ng/μL dissolved Aβ40, Aβ42, Aβ40-1, and Aβ42-1 for 16 h. Recombinant cystatin F dimers were added to the wells for 3 h. The primary antibody for cystatin F and the secondary antibody coupled with HRP were used to generate a colour reaction, and the absorbance was measured at 450 nm. **C**, **D** The GST pull-down assay and His pull-down assay were used to analyse the interaction between the cystatin F dimer and Aβ. **E** Schematic diagram depicting the BiFC assay for detecting the interaction between the cystatin F dimer and Aβ. **F** The BiFC assay was carried out as described in the Materials and Methods section. Then, 200 ng/mL cystatin F dimer-VN and 200 ng/mL Aβ-VC protein were incubated at 37 °C for 2 h, and the fluorescence intensity was assessed at 529 nm. **G** Prediction of the amino acid interactions between cystatin F dimer and Aβ. **H-J** The potential amino acids on cystatin F that interacted with Aβ were mutated to alanine. The interaction was determined by a GST pull-down assay. **K**–**L** THP-1 cells were pretreated with 250 ng/mL cystatin F dimer protein or cystatin F (RTSTK) mutant protein for 30 min, 1 μg/mL Aβ42-Alexa Fluor 647 or Aβ42 was added for an additional 30 min, and the cells were subjected to **K** FCM and **L** ELISA analysis**. M**, **N** THP-1 cells were pretreated with 250 ng/mL cystatin F dimer protein or cystatin F (RTSTK) mutant protein for 30 min, and 1 μg/ml Aβ42-Alexa Fluor 555 or Aβ42-Alexa Fluor 647 were added for an additional 30 min, and the cells were observed by **M** TIRFM and subjected to **N** FCM**.** The data are presented as the means ± SD and were analysed using Student’s t test and one-way ANOVA. ^**^p < 0.01, ^***^p < 0.001. Scale bar: 5 μm

High concentrations of the cystatin F dimer present with Aβ in the circulation and inhibit the internalization of Aβ by monocytes; therefore, we first investigated whether there is a potential relationship between them. Cystatin F shares a high homology with cystatin C, which belongs to the same family and can interact with Aβ [56]. We explored the potential associations, assuming that dimeric cystatin F binds to Aβ. A binding assay that can estimate the binding capacity of the two proteins showed that the cystatin F dimer protein directly binds to Aβ40 and Aβ42 in a dose-dependent manner (Fig. 5A, B). Both GST and His pull-down assays further demonstrated a strong interaction between the cystatin F dimer and Aβ (Fig. 5C, D). Furthermore, we used BiFC to investigate how the cystatin F dimer interacts with Aβ in vitro. After tagging the two proteins at their N-terminal and C-terminal fragments of fluorescent Venus protein (Fig. 5E), the cystatin F dimer-VN protein and Aβ-VC protein were expressed in 293T cells and purified with a nickel agarose column. When the purified cystatin F dimer-VN protein was incubated with the Aβ-VC protein, the fluorescence intensity was measured at 529 nm, and the results indicated that the cystatin F dimer is capable of binding to Aβ (Fig. 5F). We further investigated whether the interaction between the cystatin F dimer and Aβ led to the inhibition of the recruitment and internalization of Aβ. We predicted the amino acids that interact with the cystatin F dimer and Aβ42, and three potential binding mechanisms were identified by the Cluspro1, HDOCK2, and MOE3 methods (Fig. 5G and sTable 3). The predicted amino acids on the cystatin F dimer that are potentially capable of interacting with Aβ were mutated to alanine, namely, cystatin F (KSRKKKWWRK), cystatin F (RTSTK) and cystatin F (YNR). GST pull-down assays revealed that cystatin F (RTSTK) had a weaker ability to bind to Aβ42 than did cystatin F (KSRKKKWWRK) and cystatin F (YNR) (Fig. 5H–J), indicating that the Thr41, Ser73, Arg77, Lys119, and Thr121 residues in cystatin F form hydrogen bonds and that salt bridges with the Asp7, Tyr10, His14, Gly33 and Leu34 residues in Aβ42 are involved in these interactions (sFig. 3). Moreover, compared with the cystatin F dimer protein, the cystatin F (RTSTK) protein or supernatant of 293T cells transfected with the cystatin F (RTSTK) plasmid restored Aβ uptake according to FCM and ELISA (Fig. 5K, L and sFig. 4A). In addition, we observed that monocytes pretreated with the cystatin F (RTSTK) mutant protein had an enhanced ability to recruit Aβ compared to monocytes pretreated with the wild type (WT) cystatin F dimer protein through TIRFM and LSCM (Fig. 5M and sFig. 4B). In parallel, the FCM analysis results showed that the adhesion of Aβ to monocytes that were pretreated with the cystatin F (RTSTK) protein was significantly improved (Fig. 5N). These results implied that the complex formed by the interaction of the cystatin F dimer and Aβ extracellularly is the major contributor to the failure of monocytes to effectively recruit and internalize Aβ.

### Tail vein injection of recombinant cystatin F dimers aggravates Aβ deposition and cognitive impairment in 5XFAD mice

**Fig. 6:**
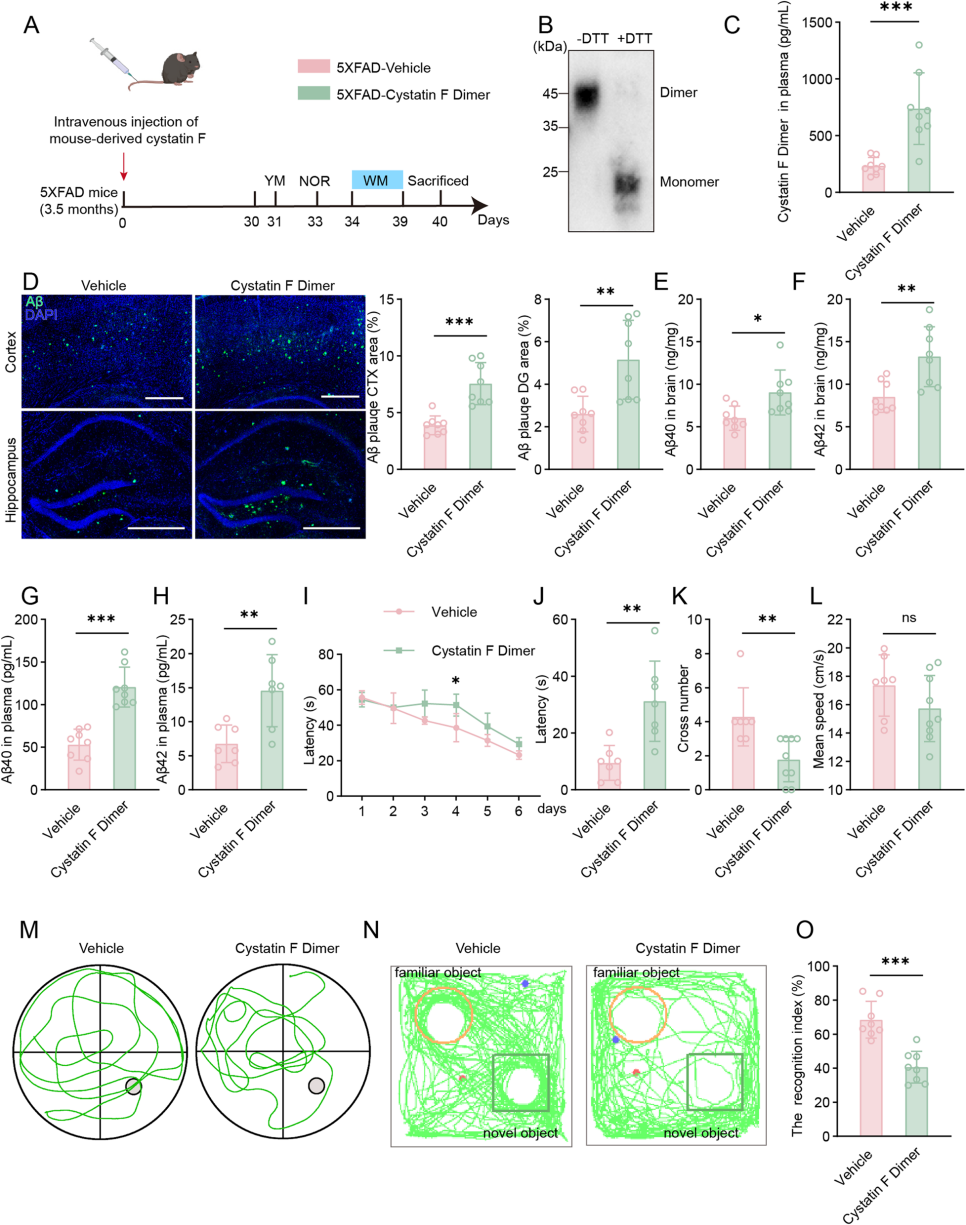
High-level cystatin F dimers in plasma rapidly aggravate cognitive impairment in 5xFAD transgenic mice. **A** Schematic of tail vein injection of 5xFAD mice, behavioural tests, and pathological analysis. **B** The purified murine cystatin F dimer was identified by western blot analysis. **C** The level of cystatin F in the plasma of the mice after tail vein injection of purified protein which was detected by ELISA analysis (n = 8). **D** Immunofluorescence and statistical analysis of Aβ plaques in brain section from transgenic mice that received a tail vein injection. Scale bar: 500 μm **E**, **F** ELISA method for measuring Aβ40 and Aβ42 levels in the brain of the mice. **G**, **H** ELISA method for measuring Aβ40 and Aβ42 levels in the plasma of the mice. **I**–**L** MWM analysis of the latency (s), number of target crosses, and mean speed (cm/s) in the invisible platform test of the mice. **M** Representative images of the track plots in the MWM test. **N** The motion traces of the NOR test were displayed.** O** The recognition index was calculated as described in Material and Methods. The data are presented as the means ± SD and were analysed using Student’s t test. ^*^p < 0.05, ^**^p < 0.01, ^***^p < 0.001

Due to the increased expression of the cystatin F dimer in a collection of patients with sporadic AD, we further explored whether cystatin F is associated with mutations in AD-related genes. We detected the expression of cystatin F in 5XFAD transgenic mice, which express human APP and PSEN1 transgenes with a total of five AD-linked mutations, namely, the Swedish (K670N/M671L), Florida (I716V), and London (V717I) mutations in APP and the M146L and L286V mutations in PSEN1 [57]. As shown in sFig. 5A, the expression of the cystatin F protein in the plasma of 5XFAD transgenic mice did not significantly increase compared with that in the plasma of control mice. Then, the 5XFAD transgenic mice, which exhibited many AD-related phenotypes at a relatively early stage, received a high dose of purified mouse cystatin F dimer via the tail vein to disperse into the peripheral blood (Fig. 6A–C). We first assessed Aβ levels from the brain to the periphery. In the brain, Aβ plaque coverage within the cortex and hippocampus significantly increased (Fig. 6D), and the levels of Aβ40 and Aβ42 markedly increased in 5XFAD-cystatin F dimer mice (Fig. 6E, F). The ELISA results showed that the levels of soluble Aβ40 and Aβ42 was increasing in the plasma of 5XFAD-cystatin F dimer mice compared with the control mice (Fig. 6G, H). Next, we investigated whether blood-borne cystatin F dimers affect central Aβ clearance in the brain. Through the detection of His tag-fused recombinant cystatin F proteins in brain sections, we found that circulating high-level cystatin F failed to diffuse into the brain (sFig. 5B) and that microglia were not affected by increased peripheral levels of cystatin F in 5XFAD mice, either morphologically or in terms of numbers (sFig. 5C). Additionally, we used a human brain microvascular endothelial cell (HBMEC) monolayer cultured on a Transwell insert by adding Aβ to the lower chamber to mimic the AD blood brain barrier model in vitro [58]. We found that monocyte transendothelial migration was not altered by elevated levels of the cystatin F dimer (sFig. 5D and E). These results suggested that high levels of the cystatin F dimer influence Aβ metabolism mainly in the periphery.

Furthermore, various behavioural tests were carried out to assess the effect of the cystatin F dimer protein on learning and memory in 5XFAD mice. The MWM results showed that 5XFAD-cystatin F dimer mice exhibited longer escape latencies during the training trials and fewer platform crossings but no significant alterations in swimming speed (Fig. 6I–M). In the NOR test, 5XFAD-cystatin F dimer mice spent less time exploring new objects, as evidenced by a significant decrease in the recognition index (Fig. 6N, O). Compared with those of the controls, the time, ambulation, and number of visits in the novel arms were obviously decreased in the 5XFAD-cystatin F mice during the Y maze tests (sFig. 5F–I). These results indicated that a high plasma cystatin F dimer level could rapidly exacerbate Aβ deposition and a memory deficit in 5XFAD mice as a risk factor.

## Discussion

The mechanism underlying the dysfunctional peripheral clearance of Aβ by monocytes derived from AD patients is unclear. Herein, we demonstrated that aberrantly elevated expression of the cystatin F dimer released by AD monocytes is a determinant of dysfunctional Aβ clearance in the periphery and contributes to AD pathology.

Monocytes and macrophages are capable of physiologically removing circulating Aβ that originates in the brain through multiple means. Similar to glial cells in the brain, monocytes/macrophages in the periphery express phagocytic receptors for Aβ and produce Aβ-degrading enzymes to recognize and degrade Aβ [59, 60]. Mounting evidence suggests that gene expression changes in peripheral immune cells can affect brain function and animal behaviour [61]. We demonstrated that cystatin F expression was specifically elevated in the monocytes of sporadic AD patients and that in vivo, human cystatin F aggravated Aβ deposition in the brain and cognitive impairment in APP/PS1 mice. Cystatin F was first identified in samples from AD patients by Keren-Shaul H et al. [62] and was reported to be significantly upregulated in microglia as an amyloid plaque’s indicator [63]. Cystatin F exhibited similarly elevated expression and functions in contributing AD development in both peripheral and central innate immune cells. This may be a result of meningeal macrophages penetrating the brain during embryonic development to form microglia, and even brain parenchymal microglia could also be derived from blood monocytes, which are regarded as the counterparts of microglia in the periphery [64, 65]. Our findings verified the importance of defects in peripheral monocytes in AD progression and that cystatin F may be a potential risk factor.

Bone marrow-derived monocytes have been studied often in AD for their ability to infiltrate the brain and engage in the phagocytosis of Aβ [66]. Since cystatin F is unable to cross the BBB and has no impact on resident microglia and transendothelial migration of monocytes, it may play a major role in Aβ clearance in the periphery. We determined that cystatin F was released as a dimer into the plasma. Additionally, the cystatin F dimer specifically impaired Aβ internalization by hindering its adhesion to the surface of monocytes. This finding not only confirms that abnormal levels of cystatin F in plasma can directly affect the phagocytosis of Aβ by monocytes but also challenges the previous view that cystatin F is inactive in its dimeric form until it is reduced into monomers [67].

Cystatin F belongs to the type II cystatin gene family, which shares approximately 38% homology with its family member cystatin C [68]. The role that Cystatin C plays in AD progression is controversial, however Cystatin C has been found to inhibit the expression of cathepsin B to degrade Aβ [69]. Moreover, cystatin C can bind to Aβ to inhibit further oligomerization and fibrosis of Aβ to protect against neuronal cell death and limit the course of AD [70, 71]. Although cystatin F is especially expressed in the microglia around Aβ plaques [72] and is regarded as a sensitive indicator of Aβ plaques in AD [31], the link between cystatin F to Aβ has not been explored. We found that the cystatin F dimer is able to physically interact with Aβ through specific amino acids. Among these amino acids, arginine and lysine, which are located at positions 77 and 119, respectively, are also found in the homologous region of cystatin F and cystatin C. The direct interaction between the cystatin F dimer and Aβ is the dominant force that inhibits the recognition and internalization of Aβ by monocytes, as weakening the interaction by destruction of the interacting amino acids restored the uptake of Aβ by monocytes. Several secreted proteins are able to alter the metabolism of Aβ by binding to Aβ in the extracellular fluid. For instance, β2M markedly accelerated Aβ42 aggregation and oligomer formation by coaggregation with Aβ [73]. Whether the binding of cystatin F to Aβ results in structural changes that affect Aβ recognition by pattern receptors on monocyte membranes is worth exploring in depth.

Sporadic AD is a progressive disorder that typically starts in midlife, decades before symptom onset. Aβ accumulation precedes the onset of other AD pathologies and a decrease in cognitive impairment [2]. Strategies targeting Aβ are thus promising disease-modifying approaches for the treatment and prevention of AD [74]. Cystatin F dimers especially rapidly exacerbated the deposition of Aβ in the brain and cognitive deficits in 5XFAD transgenic mice, suggesting that this protein is a promising target in peripheral blood. An important reason for the uncontrollability of AD progression is the lack of early noninvasive diagnostic biomarkers. Considerable research has focused on changes in certain signature proteins of AD in the plasma because these proteins are easily accessible and noninvasive [75, 76]. The increase in the cystatin F dimer level in plasma from patients with AD patients was closely correlated with the Aβ level and clinical mental score of patients with AD, suggesting that the cystatin F dimer level in plasma from patients with AD might be useful for assessing AD severity.

There are several limitations to this study. First, the interaction of the cystatin F dimer with Aβ likely occurs through multiple amino acids; thus, it is difficult to use genetic or pharmacological approaches to disrupt this interaction to enhance the recognition and phagocytosis of Aβ by monocytes. Second, sex differences exist in clinical AD, and preclinical model animals also exhibit sexual dimorphisms in AD-like behaviours. In particular, Daniels MJD et al. recently reported that cystatin F plays a sexually dimorphic role in regulating microglia in *Cst7*-deficient AD mice and that the microglia of female mice have a greater Aβ burden in vivo [77]. To avoid the potential confounding effects of sex, only male mice were used in this study. Thus, our findings in male mice may not be completely recapitulated in female mice. Finally, although cystatin F may be a potential target, reducing or eliminating cystatin F from the periphery is a challenge for the future.

## Conclusions

Our study revealed the pathophysiological importance of elevated circulatory cystatin F expression in the periphery of AD patients. Specifically, we showed that cystatin F expression is increased in the peripheral monocytes of AD patients, exacerbating Aβ deposition in the brain and the pathogenesis of AD. Most interestingly, cystatin F in the plasma of AD patients exists as a dimer and is closely correlated with the severity and clinical manifestations of AD. Moreover, we noted that the cystatin F dimer blocks the phagocytosis of Aβ by monocytes by interacting with Aβ and, in vivo, aggravates Aβ deposition and cognitive dysfunction in AD model mice. Overall, our findings highlight that the cystatin F dimer plays a crucial role in regulating Aβ metabolism in the peripheral clearance pathway, providing us with a potential biomarker for diagnosis and a potential target for therapeutic treatment.

### Supplementary Information

**Additional file 1.** Supplementary figures and tables.

s

## Data Availability

All data generated or analysed during this study are included in this published article and its supplementary information files. The datasets produced during the current study are also available from the corresponding author upon reasonable request.

## Abbreviations

AD: Alzheimer’s disease
APP: Amyloid precursor protein
AUC: Area under the curve
BiFC: Bimolecular fluorescence complementation
CBB: Coomassie brilliant blue
CDR: Clinical dementia rating
DAM: Disease-associated microglia
DAPI: 4′,6-Diamidino-2-phenylindole
DiOC18(3): 3,3′-Dioctadecyloxacarbocyanine perchlorate
DTT: Dithiothreitol
ELISA: Enzyme-linked immunosorbent assay
FC: Fold change
FCM: Flow cytometry
GAPDH: Glyceraldehyde-3-phosphate dehydrogenase
GO: Gene Ontology
GSEA: Gene set enrichment analysis
HBMEC: Human brain microvascular endothelial cell
Hmo-cys F^+^: Human cystatin F under the control of the human CD68 promoter
HRP: Horse radish peroxidase
LSCM: Laser scanning confocal microscope
MMSE: Minimum mental state examination
MoCA: Montreal cognitive assessment
MW: Molecular weight
MWM: Morris water maze
NINCDS-ADRDA: The National Institute of Neurological and Communication Disorders and the Stroke and Alzheimer’s Disease and Related Disorders Association
NOR: Novel object recognition
ORF: Open reading frame
PBS: Phosphate buffered saline
PEI: Polyethylenimine
PFA: Paraformaldehyde
RAVLT: Rey auditory verbal learning test
ROC: Receiver operating characteristic
RT-qPCR: Real-time polymerase chain reaction
TIRFM: Total internal reflection fluorescence microscopy
WT: Wild type
