# Supplementary material for: Monocytes release cystatin F dimer to associate with Aβ and aggravate amyloid pathology and cognitive deficits in Alzheimer’s disease
Source: J Neuroinflammation. 2024 May 10;21:125. doi: 10.1186/s12974-024-03119-2 (PMC11088181; doi:10.1186/s12974-024-03119-2)
Supplement: Supplementary file 1 — Additional file 1. Supplementary figures and tables. [file 12974_2024_3119_MOESM1_ESM.docx]

**Supplemental Information**

**Materials and Methods**

**Plasmid construction and transfection**

The full-length ORF of WT cystatin F or cystatin F (RTSTK) mutant was subcloned into pcDNA3.1(+)/myc-His A plasmid. The recombinant plasmids were transfected into 293T cells with PEI (Polysciences, IL, USA, Cat No. 02371-500) at a ratio of 1 µg of DNA to 2.5 µg of PEI, and cells were conﬁrmed by western blot analysis. The supernatant was collected and added into the THP-1 cells preparing for the internalization assay.

**Internalization assay**

For the flow cytometry analysis (FCM) , 3×10^5^ cells per well were cultured in 24-well plates, the cystatin F dimer protein was added to the plates at final concentrations of 250 ng/mL for 30 min, and then, Fransferrin-Alexa Fluor 488 (Jackson ImmunoResearch, PA, USA, Cat No. 009-090-050) and Dextran-Alexa Fluor 647 (Invitrogen, CA, USA, Cat No. D22914) at a final concentration of 1 μg/mL, was added for a further 30 min of incubation at 37°C, after which the cells were subjected to FCM analysis.

For the uptake inhibition assay, cytochalasin D (GLPBIO, CA, USA, Cat No. D22914GC13440) and EIPA (MedChemExpress, NJ, USA, Cat No. HY-101840) at final concentrations of 5 μg/mL and 40 μM, respectively, were added to the cells to pretreat for 30 min and 1 h, respectively. The cells were incubated with the cystatin F dimer at a final concentration of 250 ng/mL for 30 min. Dextran-Alexa Fluor 647 at a final concentration of 1 μg/mL was added to the cells, which were incubated for 30 min and analyzed via FCM. The data were analyzed with InCyte software (Millipore, Darmstadt, Germany) and visualized using FlowJo software (Tree Star, Inc., CA, USA).

**Protein-protein docking**

The protein cystatin F dimer is set as the receptor before docking, while Aβ42 is set as the ligand. The ligand was spun 70,000 times in Cluspro. On a grid, the ligand was moved in the x, y, and z axes in relation to the receptor for each revolution. From each rotation, the translation with the highest score was picked. 1000 rotation/translation combinations with the lowest scores out of 70,000 total rotations were chosen. The next step was to execute a greedy clustering of these 1000 ligand positions with a 9 C-alpha RMSD radius to identify the ligand sites with the greatest number of cluster centers. After that, the top 10 cluster centers with the greatest number of cluster members were retrieved and visually examined one by one. Using a hybrid docking approach, the HDOCK server predicts the binding complexes between two molecules such as proteins and proteins. The multi-stage process of generating postures and ranking them was applied in MOE. Exhaustive sampling is used to create a series of initial postures starting from a coarse-grained model, which shrinks the computational search area. The Hopf fibration is used to create a collection of evenly distributed rotations, and the Fast Fourier Transformis used to sample every translation for a particular rotation. A minimizing procedure structured around a staged convergence protocol comes next. The top three conformations were chosen as the final (probable) binding mode when docking was complete. The MOE Protein contacts module was used to evaluate the docked structures and interface residues. PyMOL was used to produce molecular visualizations.

**Y maze test**

The Y maze consisted of three arms with an angle of 120° between each of the arms. In brief, the first trial (training) with a 10-min duration was performed to allow the mouse to explore only two arms (start arm and familiar) of the maze, with the third arm (novel arm) blocked. The second trial (testing) was conducted after a 1 h interval; mice were placed back in the maze in the same starting arm, with free access to all three arms for 10 min. The time, ambulation, and number of visits in both the novel and familiar arms were recorded by the investigator, and the exploration time, ambulation, and number of visits in the novel arm were quantified as percentages of both the novel and familiar arms.

**Transendothelial migration assay of monocytes**

Human peripheral monocytes were separated with Dynabeads Isolation Reagent (Invitrogen, Carlsbad, CA) according to the manufacturer’s protocols. Briefly, 2 × 10^5^ HBMEC were seeded on the upper chamber of Transwell insert with 5 μm pore size (Corning Costar Corp., Cambridge, MA) in 24-well plates. The integrity of the HBMEC monolayer was monitored by daily transendothelial electrical resistance (TEER) measurements, using a Millicell-ERS endothelia volt-ohmmeter (World Precision Instruments Inc.). Experiments were conducted 4d after plating when TEER was > 200 Ω × cm^2^. Monocytes (1 × 10^6^) with 250 ng/mL cystatin F dimer protein were loaded into the upper chamber of the Transwell insert and Aβ (125nM) was added to the lower chamber. After incubation for 24h, migrated monocytes from the lower chamber were collected and counted in a hemacytometer in triplicate.

**Supporting figures**


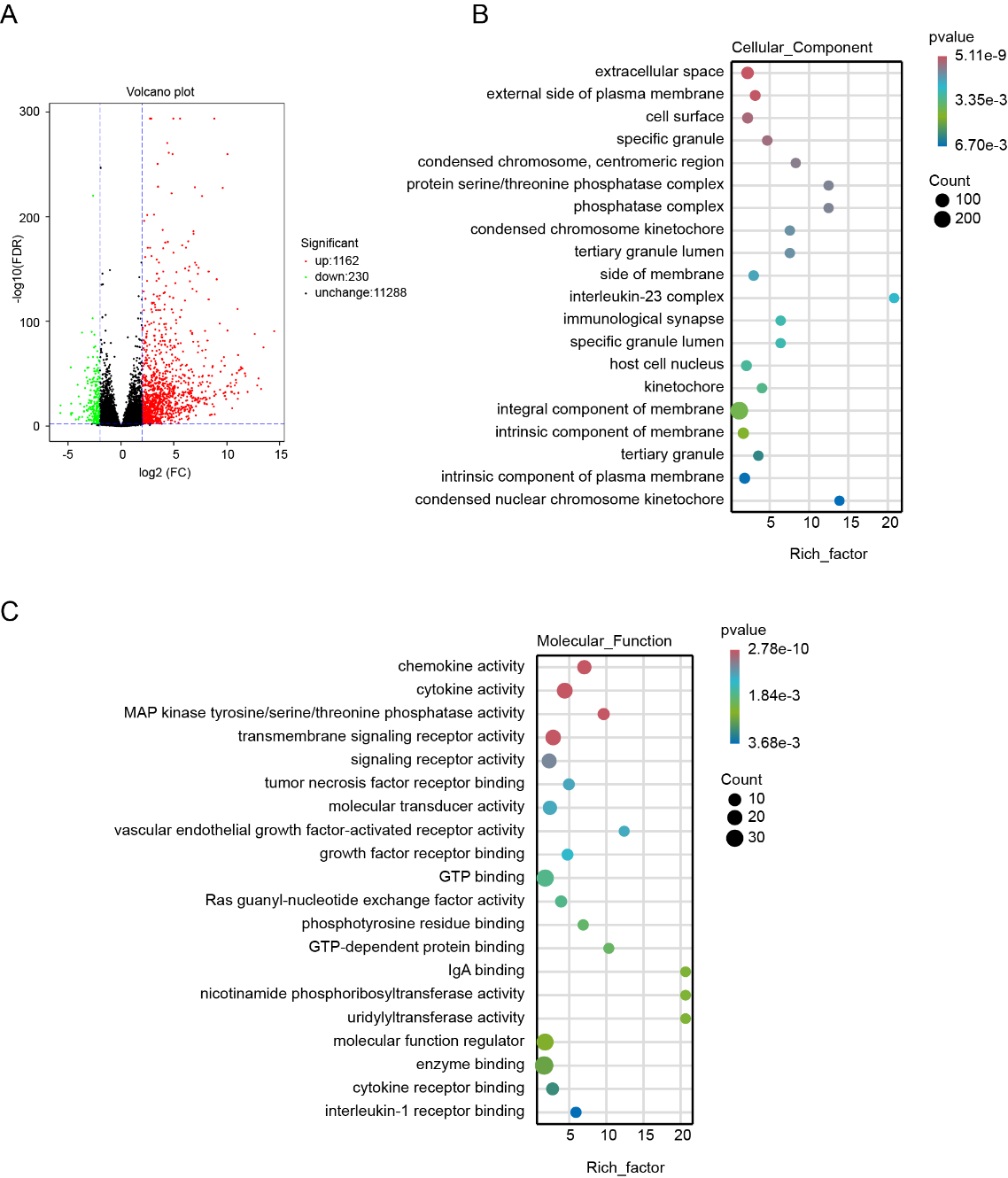


sFig 1 Bioinformatics analysis of gene ontology (GO) pathways with differential gene expression. **A** Volcanic map of differential gene expression between the AD monocytes (n=9) and age-matched controls (n=9). **B, C** GO enrichment analysis of **B** Cellular Component and **C** Molecular Function in the Top 20 signaling pathway.


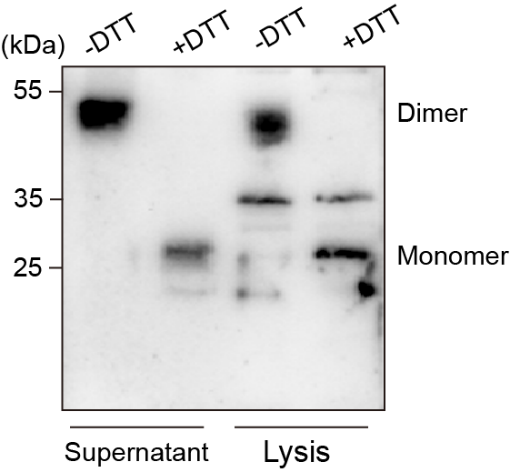


sFig 2 Identification the expression and structure of cystatin F in monocytes. The structure of cystatin F in human monocytes were detected by western-blot by using a nondenaturing gel and the DTT were added into the sample to reduce the dimers to monomers.


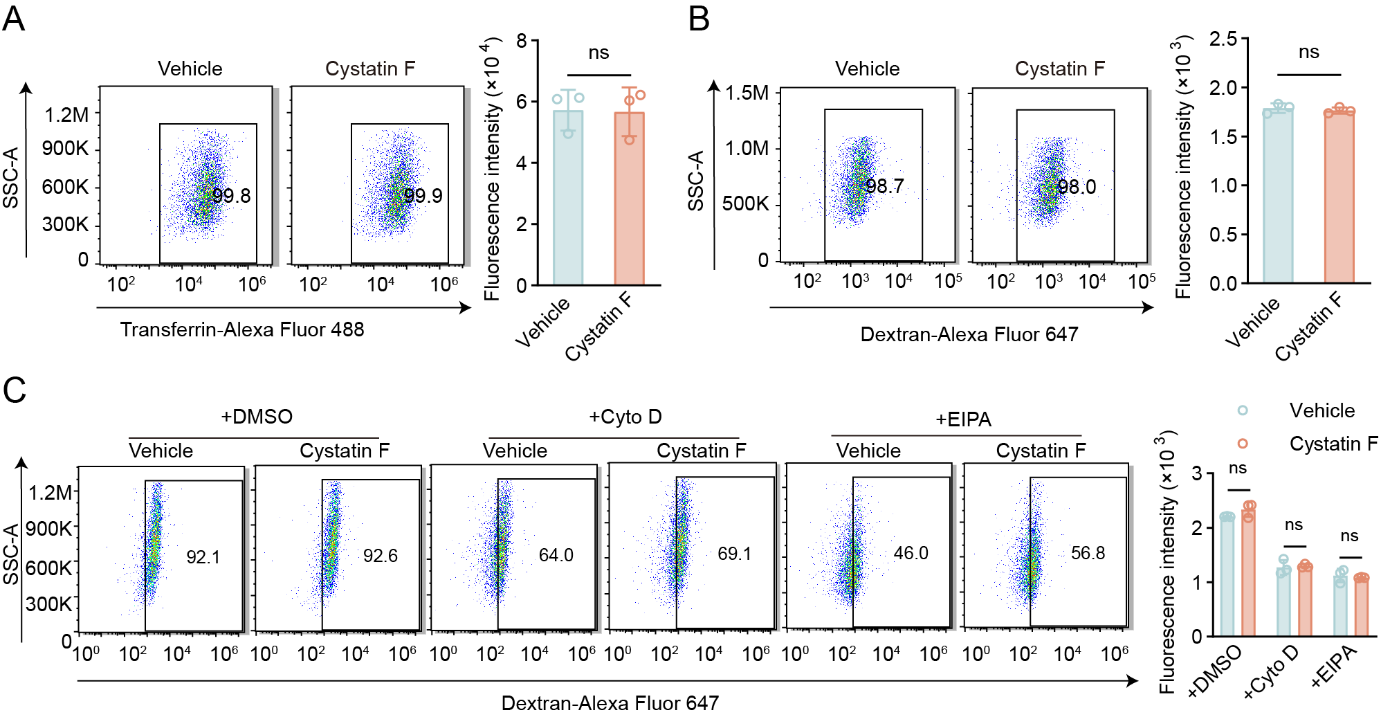


sFig 3 Cystatin F dimer does not affect the internalization of Dextran by monocytes. THP-1 cells were pretreated with 250 ng/mL cystatin F dimer protein for 30 min, and 1 μg/mL **A** Transferrin-Alexa Fluor 488 and **B** Dextran-Alexa Fluor 647 were added for an additional 30 min. FCM was performed to detect Aβ internalization. **C** THP-1 cells were pretreated with 5 μg/mL cytochalasin D for 30 min or 40 μM EIPA for 1h, and successively 250 ng/mL cystatin F for 30 min. Then 1 μg/mL soluble dextran-Alexa Fluor 647 was incubated with cells for 30 min and performed FCM analysis.


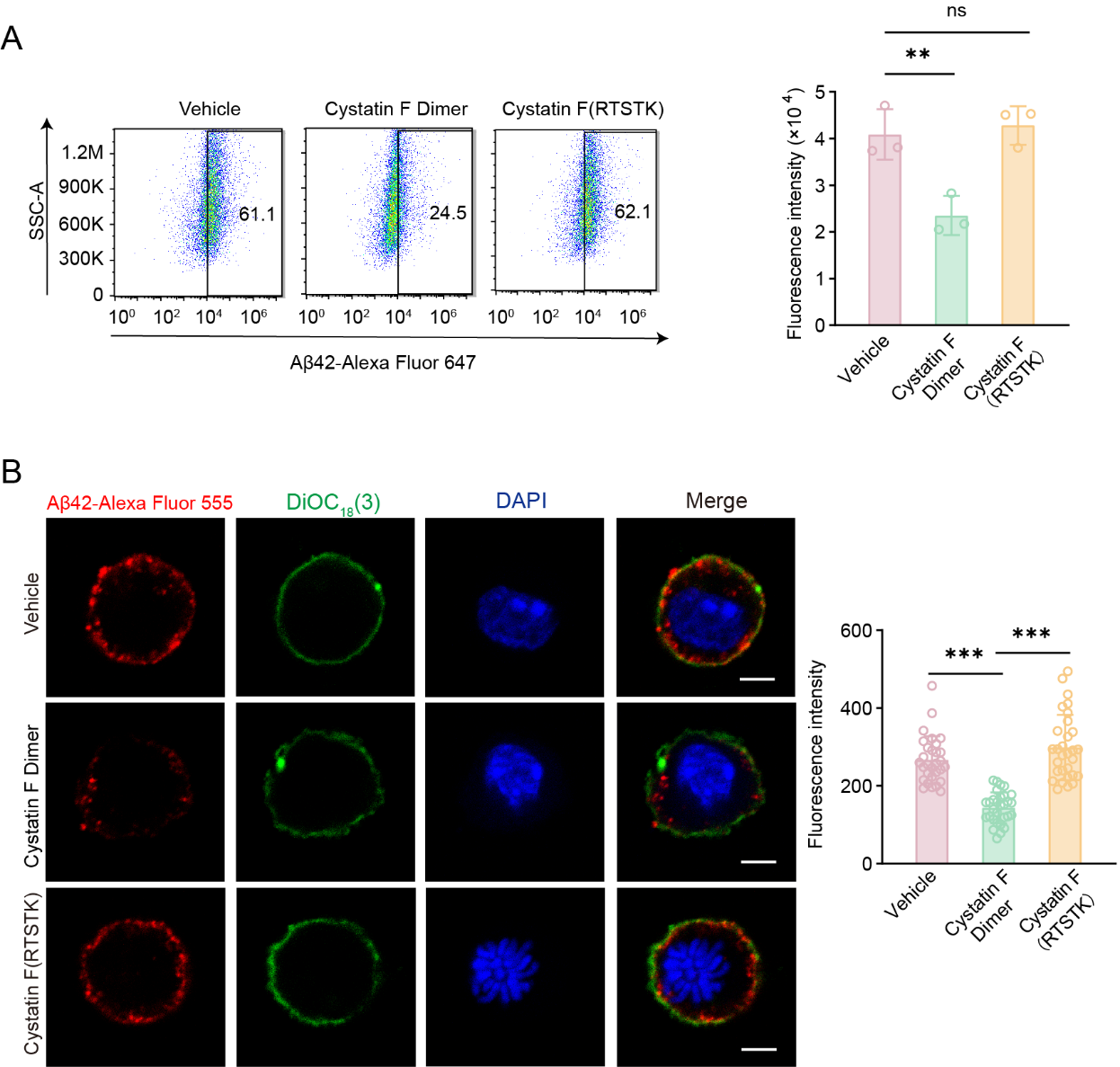


sFig 4 Cystatin F mutants inhibited the uptake and recruitments of Aβ by monocytes. **A** Cystatin F dimer or cystatin F (RTSTK) plasmid or empty vector were transfected into 293T cells and the supernatant was collected to pretreat the THP-1 cells for 30 min, then 1 μg/mL soluble Aβ42-AlexaFluor 647 was added into cells. After 30 min incubation, cells were washed three times and performed FCM analysis. **B** THP-1 cells were pretreated with 250 ng/mL cystatin F protein, and 1 μg/mL soluble Aβ42-AlexaFluor 555 was added for an additional 30 min at 0°C, then cells were observed by LSCM. Data were the mean ± SD and analyzed using a one-way ANOVA. ^**^*p* <0.01, ^***^*p* <0.001. Scale bar: 5 μm


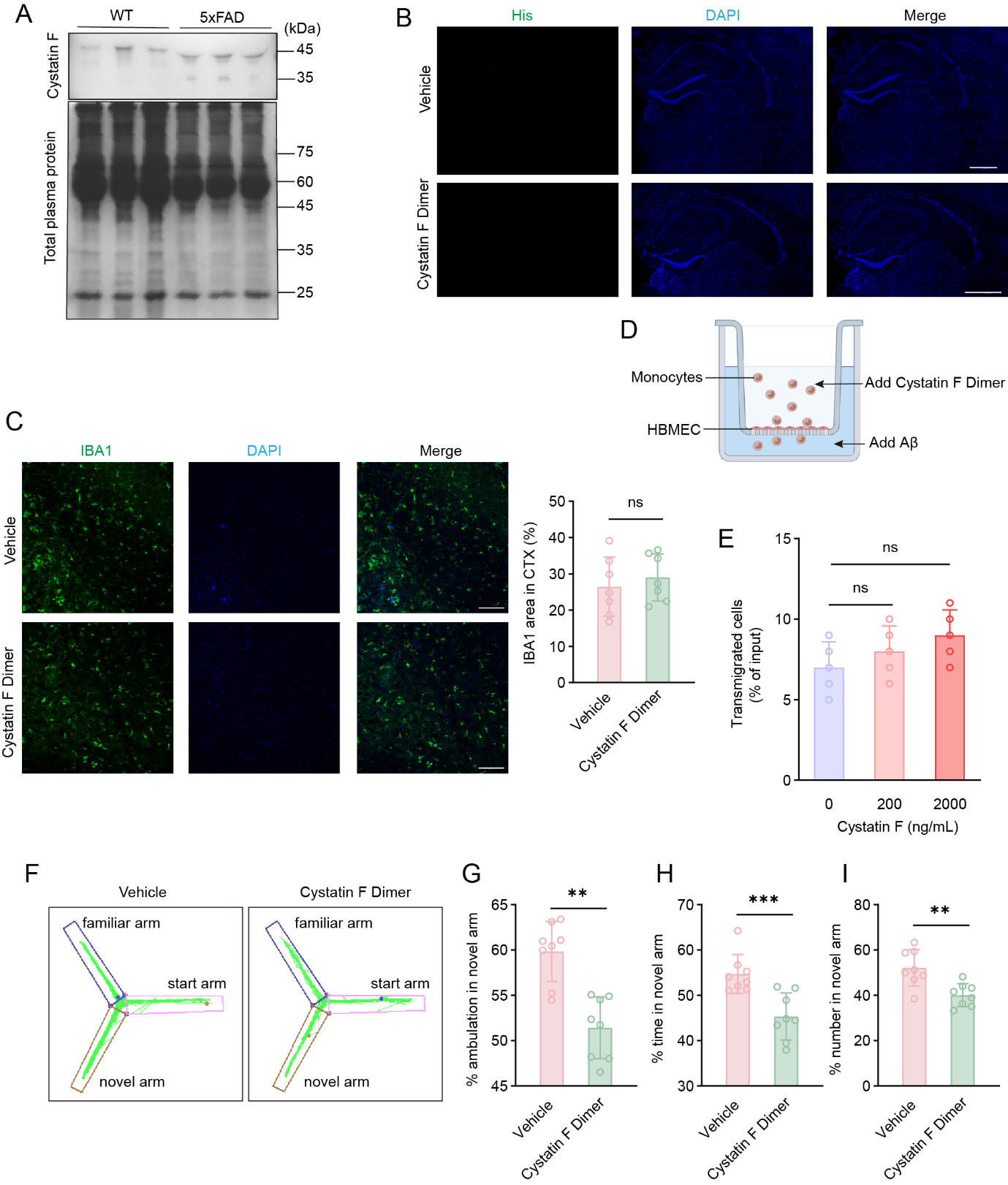


sFig 5 Cystatin F dimer in the plasma failed to entry into the brain to affect the microglia. **A** The expression of murine cystatin F protein in 5XFAD mice plasma (n=3) and controls (n=3) were detected by western-blot by using a nondenaturing gel. **B** Immunofluorescence detection of His-cystatin F fusion protein in the brain slices of 5XFAD mice by using anti-His antibody. Scale bar: 500 μm. **C** Morphological and quantitative analysis of microglia in 5XFAD mice which received the purified mouse cystatin F protein via tail vein injection. Scale bar: 20 μm. **D, E** Transendothelial migration assays were performed in the presence of Aβ in 125nM in the lower chamber and cystatin F dimer in 200ng/mL or 2000 ng/mL in the upper chamber of the Transwell insert cultured with HBMEC. Monocytes were added to the upper chamber of Transwell insert for 24 h. Transmigrated cells were harvested and counted. The results represent three independent experiments. Data were the mean ± SD. **F-I** The Y maze test was performed to assess the spatial memory of the mice. The mice were trained for 10 min in both start and familiar arms for 1 h. The mice were returned to the maze at the starting arm, with free access to all three arms, and were allowed 10 min to explore the maze. The representative motion tracks of the test session were provided. ^**^*p* <0.01. ^***^*p* <0.001.

| **sTable 1. Characteristics of study subjects in the screening and validation stage** | | | | | |
| --- | --- | --- | --- | --- | --- |
| Characteristics | Microarray analysis | | Validation | | |
|  | Control | AD | Control | AD | |
| Enrollment time | 5 months | 5 months | 36 months | | 36 months |
| Total number | 9 | 9 | 40 | | 40 |
| Age (mean ± SD) | 63.2±6.2 | 61.7±7.6 | 63.23±7.8 | | 63.7±8.6 |
| Sex (Male/Female) | 4/5 | 2/7 | 20/20 | | 16/24 |
| Education (mean ± SD) | 10.8±1.4 | 10.3±2.7 | 10.7±0.4 | | 10.8±1.7 |
| MMSE (mean ± SD) | 28.43±3.6 | 17.7±7.2 | 27.21±1.6 | | 11.3±6.3 |
| MoCA (mean ± SD) | 25.45±5.3 | 13.0±7.2 | 26.05±1.3 | | 7.0±4.9 |
| RAVLT-I (mean ± SD) | 28.76±7.5 | 10±5.5 | 27.34±2.5 | | 6.0±5.6 |
| RAVLT-D (mean ± SD) | 12.34±2.3 | 0.9±1.4 | 13.22±1.1 | | 0.3±0.9 |
| CDR (mean ± SD) | 0±0 | 1.2±0.8 | 0±0 | | 2.0±0.8 |

| **sTable 2. Specificity analysis of differentially expressed genes** | | | | | |  |
| --- | --- | --- | --- | --- | --- | --- |
| **Gene symbol** | **Protein** | **log_2_FC** | **FDR** | **RNA immune Specificity** | **Cell line distribution** | |
| IL12B | Interleukin 12B | 11.54099931 | 3.66E-51 | Non-specific | Lymphoma - Immune response | |
| SUCNR1 | Succinate receptor 1 | 5.498734223 | 1.21E-16 | Specific | Rhabdoid | |
| EBI3 | Epstein-Barr virus induced 3 | 3.43006979 | 2.40E-21 | Specific | Lymphoma - Immune response | |
| IL18RAP | Interleukin 18 receptor accessory protein | 2.787242402 | 4.61E-08 | Non-specific | Lymphoma - Inflammatory response | |
| CST7 | Cystatin F | 2.341606599 | 6.52E-18 | Specific | Myeloid leukemia - Innate immune response | |
| MMP25 | Matrix metallopeptidase 25 | 2.175651828 | 2.70E-22 | Specific | HMC-1 - Innate immune response | |

**sTable 3. The contact list between Aβ (1-42) and CST7 dimer.**

| Pose | Chain 1 | Residue | Chain 2 | Residue | Interaction type |
| --- | --- | --- | --- | --- | --- |
| Pose1 | CST7.A | Lys43 | Aβ (1-42) | Gly38 | Hydrogen bond interaction |
| Pose1 | CST7.B | Ser32 | Aβ (1-42) | Tyr10 | Hydrogen bond interaction |
| Pose1 | CST7.B | Arg33 | Aβ (1-42) | His6 | Hydrogen bond interaction |
| Pose1 | CST7.B | Lys43 | Aβ (1-42) | Asn27 | Hydrogen bond interaction |
| Pose1 | CST7.B | Lys43 | Aβ (1-42) | Lys28 | Hydrogen bond interaction |
| Pose1 | CST7.B | Lys84 | Aβ (1-42) | Gln15 | Hydrogen bond interaction |
| Pose1 | CST7.B | Trp133 | Aβ (1-42) | His13 | Hydrogen bond interaction |
| Pose1 | CST7.B | Trp133 | Aβ (1-42) | His14 | Hydrogen bond interaction |
| Pose1 | CST7.B | Arg33 | Aβ (1-42) | Asp7 | Salt bridge |
| Pose1 | CST7.B | Lys40 | Aβ (1-42) | Glu22 | Salt bridge |
| Pose1 | CST7.A | Thr41 | Aβ (1-42) | Val39 | VdW |
| Pose1 | CST7.A | His145 | Aβ (1-42) | Phe4 | VdW |
| Pose1 | CST7.B | Arg33 | Aβ (1-42) | Tyr10 | VdW |
| Pose1 | CST7.B | Val34 | Aβ (1-42) | Phe4 | VdW |
| Pose1 | CST7.B | Val34 | Aβ (1-42) | Tyr10 | VdW |
| Pose1 | CST7.B | Val34 | Aβ (1-42) | Gln15 | VdW |
| Pose1 | CST7.B | Lys35 | Aβ (1-42) | Phe4 | VdW |
| Pose1 | CST7.B | Thr41 | Aβ (1-42) | Ile31 | VdW |
| Pose1 | CST7.B | Ile82 | Aβ (1-42) | Val18 | VdW |
| Pose1 | CST7.B | Ile82 | Aβ (1-42) | Ala21 | VdW |
| Pose1 | CST7.B | Ile82 | Aβ (1-42) | Glu22 | VdW |
| Pose1 | CST7.B | Leu86 | Aβ (1-42) | Ala21 | VdW |
| Pose1 | CST7.B | Trp133 | Aβ (1-42) | Gln15 | VdW |
| Pose1 | CST7.B | Trp133 | Aβ (1-42) | Val18 | VdW |
| Pose1 | CST7.B | Leu134 | Aβ (1-42) | His13 | VdW |
| Pose1 | CST7.B | Leu134 | Aβ (1-42) | His14 | VdW |
| Pose2 | CST7.A | Arg77 | Aβ (1-42) | Gly33 | Hydrogen bond interaction |
| Pose2 | CST7.B | Thr41 | Aβ (1-42) | Leu34 | Hydrogen bond interaction |
| Pose2 | CST7.B | Ser73 | Aβ (1-42) | Tyr10 | Hydrogen bond interaction |
| Pose2 | CST7.B | Thr121 | Aβ (1-42) | His14 | Hydrogen bond interaction |
| Pose2 | CST7.B | Lys119 | Aβ (1-42) | Asp7 | Salt bridge |
| Pose2 | CST7.A | Lys40 | Aβ (1-42) | Val24 | VdW |
| Pose2 | CST7.A | Ala78 | Aβ (1-42) | Leu34 | VdW |
| Pose2 | CST7.B | Lys40 | Aβ (1-42) | Gly33 | VdW |
| Pose2 | CST7.B | Lys40 | Aβ (1-42) | Leu34 | VdW |
| Pose2 | CST7.B | Lys40 | Aβ (1-42) | Gly37 | VdW |
| Pose2 | CST7.B | Lys40 | Aβ (1-42) | Val39 | VdW |
| Pose2 | CST7.B | Thr41 | Aβ (1-42) | Val36 | VdW |
| Pose2 | CST7.B | Arg74 | Aβ (1-42) | Val18 | VdW |
| Pose2 | CST7.B | Leu79 | Aβ (1-42) | Leu34 | VdW |
| Pose2 | CST7.B | Glu91 | Aβ (1-42) | Ala21 | VdW |
| Pose2 | CST7.B | Ile94 | Aβ (1-42) | Tyr10 | VdW |
| Pose2 | CST7.B | His116 | Aβ (1-42) | Ser8 | VdW |
| Pose2 | CST7.B | Gln120 | Aβ (1-42) | His14 | VdW |
| Pose2 | CST7.B | His145 | Aβ (1-42) | Leu17 | VdW |
| Pose3 | CST7.B | Tyr55 | Aβ (1-42) | Glu22 | Hydrogen bond interaction |
| Pose3 | CST7.B | Asn62 | Aβ (1-42) | Tyr10 | Hydrogen bond interaction |
| Pose3 | CST7.B | Arg106 | Aβ (1-42) | His13 | Hydrogen bond interaction |
| Pose3 | CST7.B | Arg33 | Aβ (1-42) | Ile41 | VdW |
| Pose3 | CST7.B | Val34 | Aβ (1-42) | Gly38 | VdW |
| Pose3 | CST7.B | Val34 | Aβ (1-42) | Val39 | VdW |
| Pose3 | CST7.B | Val34 | Aβ (1-42) | Ile41 | VdW |
| Pose3 | CST7.B | Gln51 | Aβ (1-42) | Ala21 | VdW |
| Pose3 | CST7.B | Ile82 | Aβ (1-42) | Leu34 | VdW |
| Pose3 | CST7.B | Ile82 | Aβ (1-42) | Gly37 | VdW |
| Pose3 | CST7.B | Ile82 | Aβ (1-42) | Gly38 | VdW |
| Pose3 | CST7.B | Ile82 | Aβ (1-42) | Val39 | VdW |
| Pose3 | CST7.B | Lys84 | Aβ (1-42) | Val39 | VdW |
| Pose3 | CST7.B | Gly85 | Aβ (1-42) | Val39 | VdW |
| Pose3 | CST7.B | Leu86 | Aβ (1-42) | Leu34 | VdW |
| Pose3 | CST7.B | His104 | Aβ (1-42) | Gly9 | VdW |
| Pose3 | CST7.B | His104 | Aβ (1-42) | Val12 | VdW |
| Pose3 | CST7.B | Arg106 | Aβ (1-42) | His14 | VdW |
| Pose3 | CST7.B | Arg106 | Aβ (1-42) | Leu17 | VdW |
| Pose3 | CST7.B | Val130 | Aβ (1-42) | Glu22 | VdW |
| Pose3 | CST7.B | Trp133 | Aβ (1-42) | Met35 | VdW |
| Pose3 | CST7.B | Trp133 | Aβ (1-42) | Val39 | VdW |
| Pose3 | CST7.B | Gln135 | Aβ (1-42) | Glu22 | VdW |
| Pose3 | CST7.B | His136 | Aβ (1-42) | Glu22 | VdW |

| **sTable 4. Primers (F, forward primer; R, reverse primer)** | | |
| --- | --- | --- |
| Application | Target gene | Primer Sequences (5′-3′) |
| RT-qPCR | cystatin F (human) | F: CTCCAAGCAGCCAGATACAG |
|  |  | R: CACGACCCAGACTTCAGAGTA |
|  | cystatin F (human)-Probe | TTCGAGGTGCCTGTTCTCCGT |
|  | GAPDH (human) | F: GAAGGTGAAGGTCGGAGTC |
|  |  | R: GAAGATGGTGATGGGATTTC |
|  | GAPDH (human)-Probe | CAAGCTTCCCGTTCTCAGCC |
| PCR | cystatin F (human) | F: CTTCCCCAGATACTTGTTCCC |
|  |  | R: AATTCCTAGTGACAACGGAGA |
|  | PSEN1 (human) | F: AATAGAGAACGGCAGGAGCA |
|  |  | R: GCCATGAGGGCACTAATCAT |
| Clone | cystatin F (human) | F: ATGCGAGCGGCTGGAACTCT |
|  |  | R: TCAGTGACAACGGAGAACAGG |
|  | cystatin F (mouse) | F: CCAAGCTGGCTAGTTAAGCTTGCCACCATGTGGCTGGCCATTCTG |
|  |  | R: GTGCTGGATATCTGCAGAATTCCTGGCAGAGGAGAACAGGC |
|  | cystatin F (human) C-terminal VN Fragment-1 | F: CCCAAGCTGGCTAGTTAAGCTTGCCACCATGCGAGCG |
|  |  | R: CCTCCACCGTGACAACGGAGAACAGGCAC |
|  | cystatin F (human) C-terminal VN Fragment-2 | F: TTGTCACGGTGGAGGCGGTTCAGG |
|  |  | R: GTGCTGGATATCTGCAGAATTCCTCGATGTTGTGGCGGATCTTG |
|  | Aβ (human) C-terminal VC Fragment-1 | F: CCCAAGCTGGCTAGTTAAGCTTGCCACCATGGATGCAGAATTCC |
|  |  | R: CCTCCACCCGCTATGACAACACCGCCC |
|  | Aβ (human) C-terminal VC Fragment-2 | F: CATAGCGGGTGGAGGCGGTTCAGG |
|  |  | R: AACTTCCAGCTTGTACAGCTCGTCCATGCC |
|  | Aβ (human) C-terminal VC Fragment-3 | F: CTGTACAAGCTGGAAGTTCTGTTCCAGGGG |
|  |  | R: GTGCTGGATATCTGCAGAATTCTCATTTACCCGGAGACAGGGAGAG |
|  |  |  |
